# The effect of antiretroviral therapy adherence on viral load suppression rate among people living with HIV in Ethiopia: A systematic review and meta-analysis

**DOI:** 10.1371/journal.pone.0358352

**Published:** 2026-09-15

**Authors:** Desalegn Dawit Assele, Yitayew Ewnetu Mohammed, Abera Gezume Ganta, Tadele Dana Darebo, Sileshi Demelash Sasie

**Affiliations:** 1 Department of Public Health, College of Medicine and Health Sciences, Hawassa University, Hawassa, Ethiopia; 2 Department of Internal Medicine, College of Medicine and Health Sciences, Hawassa University, Hawassa, Ethiopia; 3 Department of Public Health, College of Health Sciences, Jinka University, Jinka, Ethiopia; 4 School of Public Health, College of Health Sciences and Medicine, Wolaita Sodo University, Wolaita Sodo, Ethiopia; 5 Ethiopian Public Health Institute, Addis Ababa, Ethiopia; SKYDA Health Nigeria, NIGERIA

## Abstract

**Background:**

Antiretroviral therapy (ART) adherence is a key determinant of viral load suppression among people living with HIV (PLHIV). In Ethiopia, evidence on the magnitude of ART adherence and its effect on virological outcomes remains fragmented. This systematic review and meta-analysis aimed to estimate the pooled prevalence of ART adherence and viral load suppression, and to measure the association between adherence and viral suppression among PLHIV in Ethiopia.

**Methods:**

This systematic review and meta-analysis used the PRISMA checklist for systematic reviews and meta-analyses. The review protocol has been registered onPROSPERO:(CRD420251125899). PubMed, ScienceDirect, Scopus, Epistemonikos, and Google Scholar were searched. The quality of included articles has been evaluated with a Newcastle–Ottawa Scale (NOS), adapted for observational studies. A random-effects model using restricted maximum likelihood (REML) with Knapp–Hartung adjustment was used to estimate pooled prevalence and odds ratio. Heterogeneity was assessed using I^2^, τ^2^, and Cochran’s Q test.

**Results:**

A total of 39 studies were included in the final analysis. The pooled prevalence of good ART adherence was 79.4% (95% CI: 74.8%–83.4%), while the pooled viral load suppression rate was 77.5% (95% CI: 72.5%–81.8%). The pooled odds ratio showed that good ART adherence was strongly associated with viral load suppression (OR = 6.30, 95% CI: 4.84–8.19). Substantial heterogeneity was observed across studies for both adherence and viral suppression outcomes (I^2^ > 90%).

**Conclusions:**

ART adherence and viral load suppression among PLHIV in Ethiopia are relatively high but remain below global targets. Good adherence was significantly associated with virologic suppression, highlighting adherence as a critical modifiable factor for achieving optimal treatment outcomes. Strengthening adherence support interventions is essential to improve virological success and advance progress toward HIV epidemic control.

## Introduction

Human Immunodeficiency Virus (HIV) remains a major global public health challenge, particularly in resource-limited countries, with substantial morbidity, mortality, and socioeconomic impact. As of 2024, an estimated 40.8 million people were living with HIV worldwide, with a disproportionately high burden in sub-Saharan Africa, accounting for nearly two-thirds of all people living with HIV (PLHIV) and the majority of acquired immunodeficiency syndrome (AIDS) – related deaths. As of 2024, national estimates from Ethiopia indicate that over 570,000 adults are living with HIV, with a prevalence rate of approximately 0.7% and an estimated 8,400 AIDS-related deaths annually [1–4].

Advances in antiretroviral therapy (ART) over the past two decades have transformed HIV/AIDS from an almost universally fatal disease into a chronic, manageable condition, significantly improving the life expectancy of PLHIV. Moreover, the significantly reduced risk of HIV transmission due to viral suppression with the use of ART has been central to efforts to stop the HIV epidemic [5,6].

To accelerate progress toward ending the HIV epidemic, the Joint United Nations Programme on HIV/AIDS (UNAIDS) introduced the 95-95-95 targets, aiming that by 2030, 95% of PLHIV will know their HIV status, 95% of those diagnosed will receive sustained ART, and 95% of individuals on treatment will achieve viral suppression [7]. Despite considerable progress, many low- and middle-income countries (LMICs), particularly those in sub-Saharan Africa, continue to face challenges in meeting these targets. Contributing factors include fragile health systems, limited healthcare infrastructure, socioeconomic inequalities, stigma and discrimination, marginalization of key populations (e.g., sex workers and prisoners), financial constraints, and disruptions caused by public health emergencies such as armed conflict and the COVID-19 pandemic [8–16].

Among the UNAIDS targets, sustained ART use and viral suppression are fundamental to both improving individual health outcomes and reducing HIV transmission at the population level [5,7]. Achieving durable viral suppression depends largely on optimal ART adherence, commonly defined as taking at least 95% of prescribed doses. However, maintaining high adherence remains difficult in many resource-limited settings because of multiple interacting factors, including medication side effects, treatment complexity, food insecurity, mental health problems, substance use, inadequate social support, HIV-related stigma, and health system barriers such as drug stock-outs, service interruptions, and insufficient adherence counseling [17–24].

In Ethiopia, substantial efforts have been made to expand ART services and improve treatment outcomes in line with the UNAIDS 95-95-95 targets. Nevertheless, primary studies conducted across the country continue to report inconsistent estimates of ART adherence and viral load suppression, as well as varying strengths of the association between adherence and virologic outcomes. Despite the growing body of evidence, no comprehensive national synthesis has summarized the levels of ART adherence, viral load suppression, and the effect of ART adherence on viral suppression in Ethiopia. Therefore, this systematic review and meta-analysis aimed to estimate the pooled prevalence of ART adherence and viral load suppression, quantify the association between ART adherence and viral suppression, assess regional variations, and provide evidence to support national HIV treatment strategies and monitor Ethiopia’s progress toward achieving the UNAIDS 95-95-95 targets.

## Methods

### Reporting and protocol registration

We conducted this systematic review and meta-analysis to investigate the effect of antiretroviral medication (ART) adherence on viral load suppression among people living with HIV in Ethiopia. This systematic review followed the PRISMA 2020 criteria (S1 File). This systematic review was pre-registered on the PROSPERO database with an ID number of CRD420251125899.

### Data sources and searches

We conducted a comprehensive literature search across different electronic databases, including PubMed, Scopus, ScienceDirect, and Google Scholar, to ensure broad coverage of relevant studies. In addition, grey literature was identified through Ethiopian university repositories and institutional databases. The search strategy combined Medical Subject Headings (MeSH) terms and free-text keywords using Boolean operators. The key search terms included “HIV” OR “Human Immunodeficiency Virus” OR “AIDS,” combined with “antiretroviral therapy” OR “ART,” “adherence” OR “compliance,” and “viral load suppression” OR “viral suppression,” along with “Ethiopia” (S2 File). The search included studies conducted from the introduction of the Universal Test and Treat (UTT) strategy in Ethiopia to November 15, 2025, and the reference lists of included studies were manually screened to identify additional relevant articles.

### Eligibility criteria

The review question was constructed through the application of the PICO framework. The Population (P) included people living with HIV/AIDS and under ART in Ethiopia. Intervention (I): Good ART adherence, defined as ≥95% adherence (or <3 missed doses per month). Comparison (C): Poor or fair ART adherence, defined as <95% adherence (or ≥3 missed doses per month). Primary outcome (O) is viral load suppression, defined by World Health Organization standards as plasma HIV RNA < 1000 copies/mL. Study design (S) included observational studies: cross-sectional, case-control, and cohort studies evaluating the correlation between ART adherence and viral suppression using a quantitative approach. Articles excluded from the analysis were those that do not address adherence and viral suppression issues; qualitative articles; review papers; case reports; and secondary articles. Also, studies conducted in other countries without information about the results of research done specifically for Ethiopia were excluded. In case of duplicates with the same dataset, the article that contained the most information or was more recent was retained.

### Outcome measurement

ART adherence was the exposure of interest and was assessed as defined in the included studies. ART adherence was assessed by self-report and was generally operationalized as taking at least 95% of prescribed ART doses (or < 3 missed doses per month for a once-daily regimen). For the meta-analysis, adherence was categorized as good adherence and poor/fair adherence. Viral load suppression was considered when viral load copies became <1000 copies/mL of blood at least 6 months after the initiation of ART [25].

### Study selection

EndNote 21 software was used to import all identified studies, and then duplicate studies were removed. The screening process occurred in two parts, which included first screening titles and abstracts and then conducting full-text evaluations. Two independent reviewers executed the selection process while they settled their disagreement through either discussion or third reviewer consultation. The study selection process used a PRISMA flow diagram, which serves as the standard method for systematic reviews.

### Data extraction

The two reviewers conducted independent data extraction through the use of a standardized data extraction form, which ensured consistency and accurate results. The extracted data contained study characteristics (author, year of publication, and study region), methodological details (study design and setting), sample size, participant characteristics, methods used to measure ART adherence, definitions of viral load suppression, and reported effect sizes such as odds ratios or raw data for calculation. Discrepancies between reviewers were resolved through consensus.

### Quality assessment

The modified Newcastle-Ottawa Scale (NOS) for observational studies was used as the assessment tool to evaluate the methodological quality of all studies that were included in the review. This tool evaluates three key domains: selection of study participants, comparability of study groups, and outcome assessment. The studies received scores according to these criteria, which established high quality at 7 or above, moderate quality at 5–6, and low quality at below 5 [26]. Only studies with moderate to high quality were included in the primary meta-analysis to ensure the reliability of the findings.

### Data synthesis and analysis

All statistical analyses were conducted using Stata version 17. The primary effect measure was the odds ratio (OR), which evaluated viral load suppression between adherent individuals and non-adherent individuals. A random 2 × 2 data set was used to derive effect sizes that were not present in the original reports. A random-effects meta-analysis model with the restricted maximum likelihood (REML) method was used to account for between-study heterogeneity. A logit transformation was used to achieve variance stabilization for proportion-based outcomes and then back-transformed pooled estimates for outcome interpretation. Cochran’s Q test, together with the I^2^ statistic, was used to assess statistical heterogeneity, where values of 25%, 50%, and 75% represent low, moderate, and high heterogeneity, respectively [27]. The between-study variance (τ^2^) was estimated using REML, and the Knapp–Hartung adjustment was applied to provide more robust and conservative confidence intervals, particularly in the presence of substantial heterogeneity.

Meta-regression studies and subgroup analysis were carried out to investigate various sources of study heterogeneity. Sensitivity analysis was performed using the leave-one-out approach, in which each study was sequentially removed to assess its influence on the overall pooled estimate and evaluate the robustness of the findings. Publication bias was assessed through visual inspection of funnel plot symmetry and statistically evaluated using Egger’s regression test, with a p-value <0.05 indicating significant asymmetry. In the presence of publication bias, the trim-and-fill method was applied to estimate the potential impact of missing studies on the overall effect size.

## Results

### Study selection

**Fig 1:**
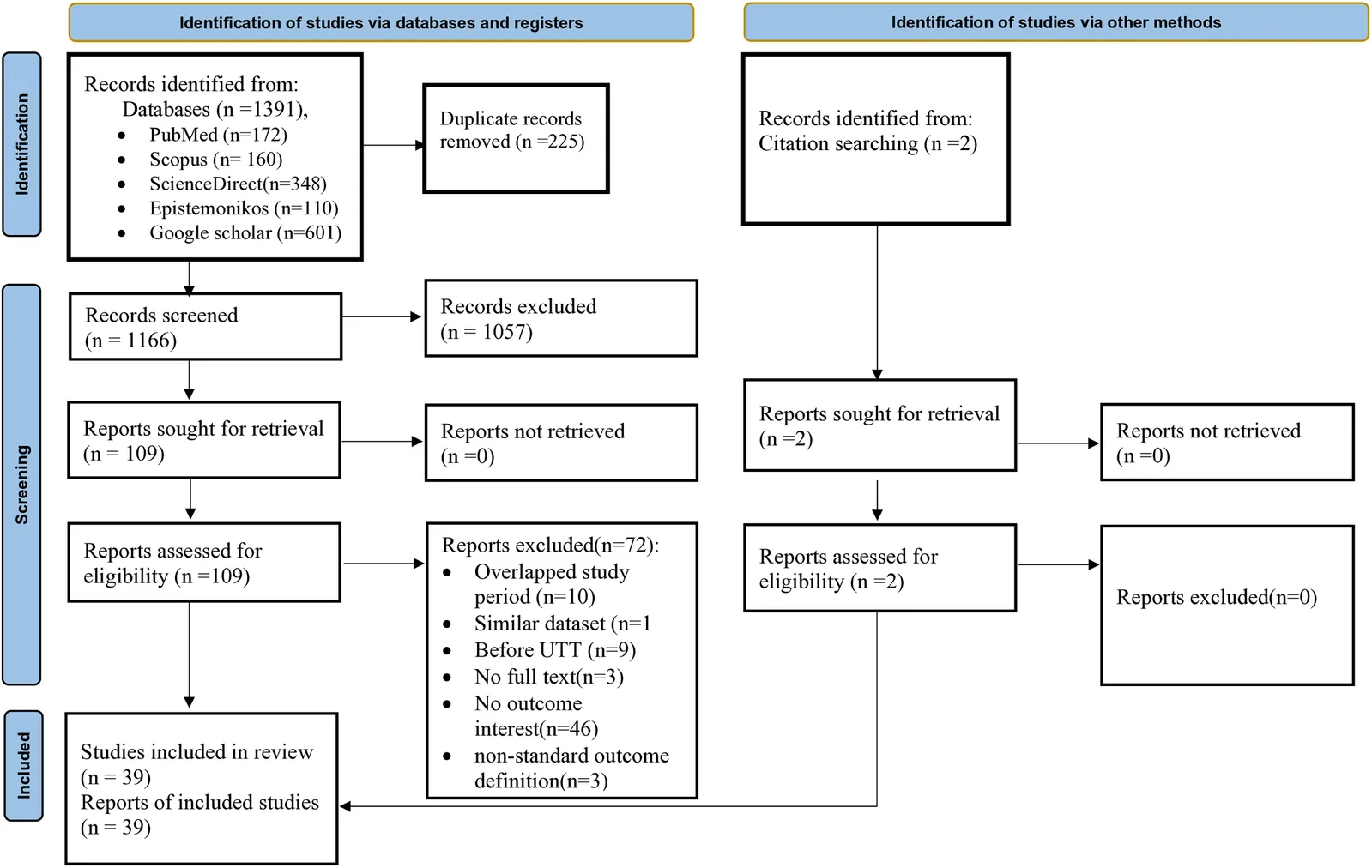
PRISMA flow diagram showing the study selection process for the systematic review and meta-analysis.

A total of 1393 studies were identified through database searching, including records from PubMed (172), Scopus (160), Epistemonikos (110), ScienceDirect (348), and Google Scholar (601), along with additional records identified through manual searches (2). After removing duplicates, 1201 unique records remained for screening. Following title and abstract screening, 109 articles were selected for full-text review. Of these, 39 studies met the inclusion criteria and were included in the final analysis. The study selection process is summarized in a PRISMA flow diagram (Fig 1).

### Characteristics of included studies

The final analysis included 39 studies conducted across different regions of Ethiopia, which included 22,412 PLWHIV. The region with the highest number of included studies were Amhara (n = 22) [28–48], Oromia (n = 6) [49–54], Addis Ababa (n = 4) [55–58], Tigray (n = 2) [59,60], and single studies from South Ethiopia [61], Southwest [62], Afar [63], South and Central Ethiopia, Sidama [64], Harar [65] and Central Ethiopia region [66]. The studies were published between 2019 and 2025, with sample sizes ranging from 117 to 7689. The majority of studies employed a retrospective cohort design (S3 File). The methodological quality of the included studies was generally satisfactory based on the Newcastle–Ottawa Scale assessment. Most studies demonstrated adequate quality in participant selection and outcome assessment domains, while some limitations were observed in the comparability domain due to insufficient adjustment for potential confounding factors. No studies were excluded based on quality assessment (S4 File).

### Pooled antiretroviral therapy adherence level

**Fig 2:**
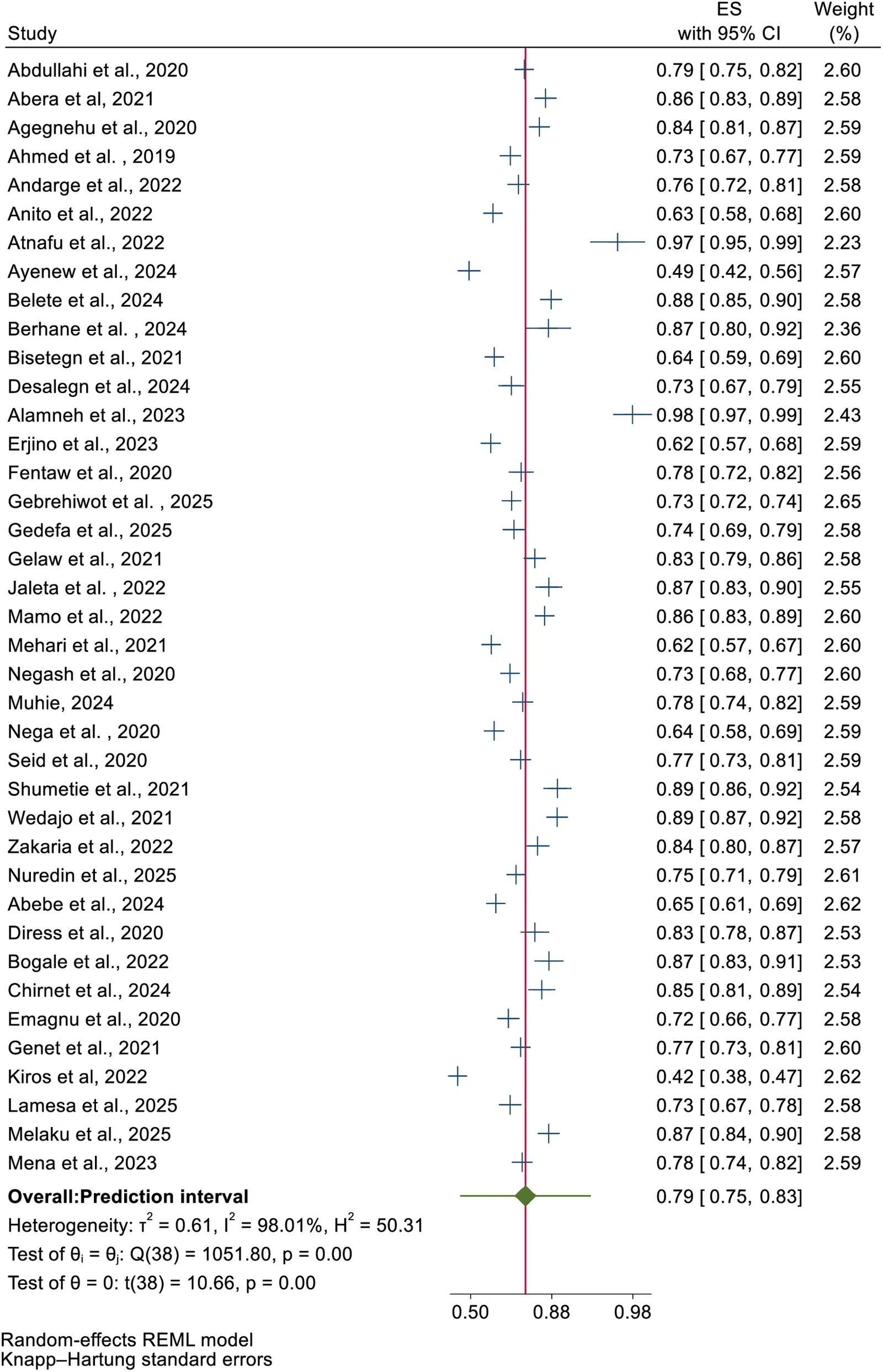
Forest plot of pooled good ART adherence among people living with HIV in Ethiopia under a random-effects model.

The pooled prevalence of good ART adherence was 79.4% (95% CI: 74.8%–83.4%). There was substantial heterogeneity among the included studies (I^2^ = 98.0%, τ^2^ = 0.60). The 95% prediction interval for the pooled adherence estimate ranged from 43.9% to 95% (Fig 2).

### Subgroup analysis of ART adherence

Pooled good ART adherence varied across subgroups, with the highest levels observed among adults (80.7%), while lower estimates were seen in the general population (73.9%). Regionally, adherence was higher in Oromia (81.7%) than in Amhara (80.2%) and Tigray (73.3%), and was greater in studies with larger sample sizes (≥500: 88.2%) than in smaller studies (76.9%). The pooled adherence among individuals receiving first-line ART was 77.2% (95% CI: 72.2%–81.5%), mixed regimens 91.0% (95% CI: 19.0%–99.8%), and it was 81.5% (95% CI: 69.0%–89.7%) among participants receiving second- and/or third-line ART (Table 1).

**Table 1:** Subgroup analysis of pooled good ART adherence among people living with HIV in Ethiopia.

Variables	Category	Number of studies	Pooled good adherence (%)	Heterogeneity within the studies	
				I2 (%)	p-value**
Population	General	14	73.9(65.5,80.8)	97.9	0.000
	Adults	18	80.7(74.6,85.7)	96.1	0.000
	Children	5	78.9(64.6,88.4)	94.8	0.000
Region	Addis Ababa	4	80.3(68.1,88.6)	82.5	0.002
	Amhara	21	80.0(71.5,86.5)	98.3	0.000
	Oromia	6	81.7(74.1,87.4)	90.5	0.000
	Tigray	2	73.3(71.0,75.5)	0.00	0.72
Sample size	<500	32	76.9(72.1,81.1)	96.3	0.000
	≥500	7	88.2(75.8,94.7)	98.8	0.000
ART regimen	First-line ART	29	77.2(72.2,81.5)	96.4	0.000
	Mixed regimens	3	91.0 (19,99.8)	99.4	0.000
	Second or/and third-line ART	7	81.5 (69.0,89.7).	96.0	0.000

### Meta-regression analysis on ART adherence

Random-effects meta-regression using REML showed no statistically significant association between publication year and ART adherence across studies (Coefficient = 0.005, p = 0.940). The model explained a negligible proportion of between-study variability (R^2^ = 0.00%), and substantial residual heterogeneity remained (τ^2^ = 0.62; I^2^ = 97.4%). Overall, there was no evidence of a temporal change in ART adherence across the included studies (S1a Fig).

### Publication bias

Visual inspection of the funnel plot indicated asymmetry, suggesting possible small-study effects (S2a Fig). This was supported by Egger’s regression test under a random-effects (REML) model, which showed significant asymmetry (p < 0.001), and Begg’s rank correlation test, which also indicated evidence of publication bias (p < 0.001). A trim-and-fill analysis using a random-effects model was conducted to assess potential publication bias in ART adherence estimates. The observed pooled proportion of good ART adherence was 79.4% (95% CI: 74.8%–83.4%), which increased to 83.9% (95% CI: 80.1%–87.2%) after adjustment for 10 potentially missing studies. This indicates that publication bias may have led to a slight underestimation of ART adherence in the original analysis; however, the adjusted estimate remained consistent, suggesting that the overall findings are robust (S3a Fig).

### Sensitivity analysis

Leave-one-out sensitivity analysis demonstrated stable pooled adherence estimates, ranging from 78.3% to 80.1%, compared with the overall estimate of 79.4% (95% CI: 74.8%–83.4%), indicating no undue influence of any single study (S4a Fig). Restricting the analysis to first-line ART studies produced a pooled adherence estimate of 77.2% (95% CI: 72.2%–81.5%), consistent with the primary analysis.

### Pooled viral load suppression rate

**Fig 3:**
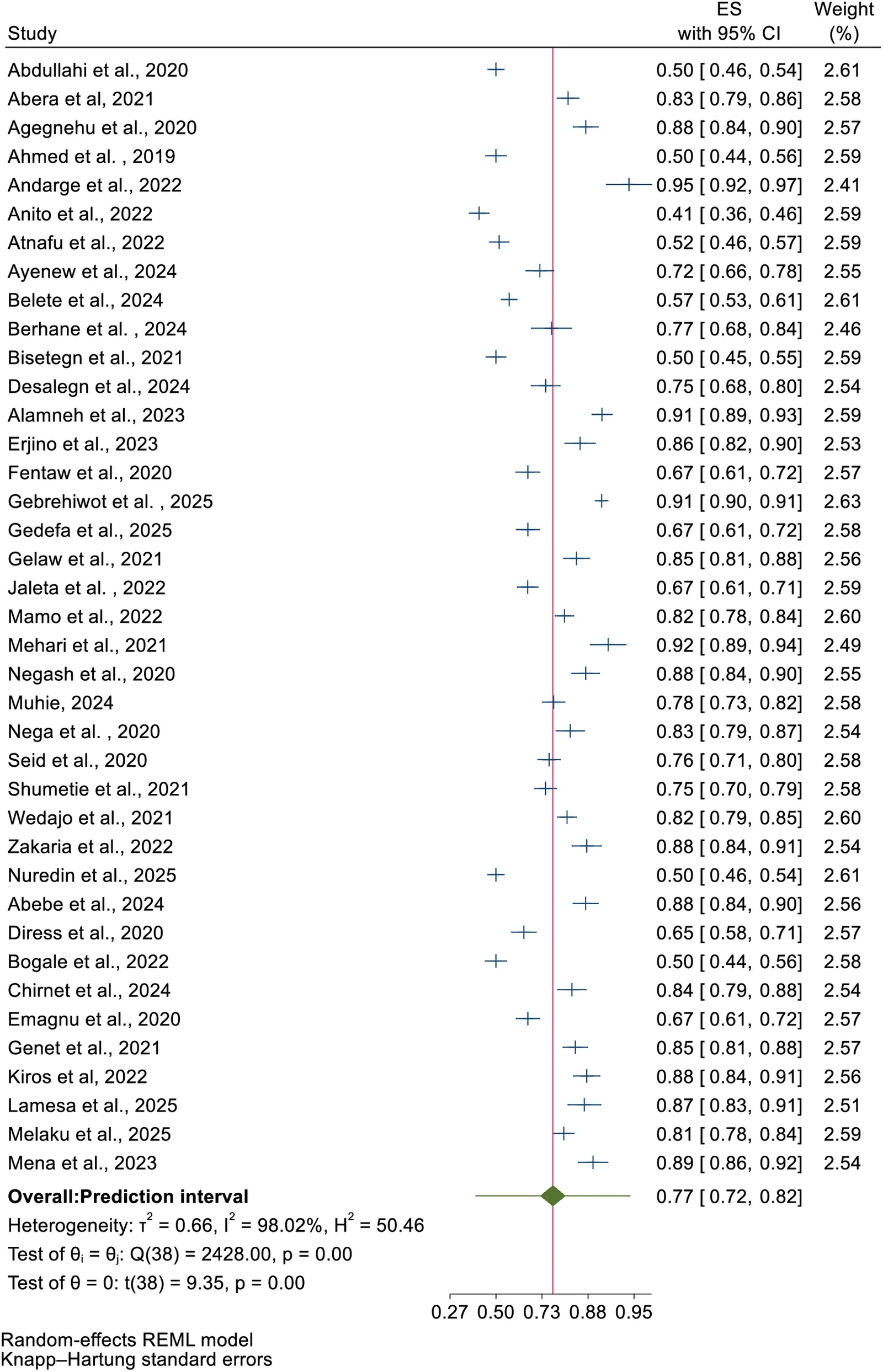
Forest plot of pooled viral load suppression among people living with HIV in Ethiopia under a random-effects model.

The pooled prevalence of viral load suppression among people living with HIV in Ethiopia was 77.5% (95% CI: 72.5%–81.8%). There was considerable heterogeneity among the included studies (I^2^ = 98%, τ^2^ = 0.66). The 95% prediction interval for the true effect size was 39.3% to 94.8%, indicating that the viral load suppression rate in a new study conducted in a similar setting could plausibly fall anywhere within this wide range (Fig 3).

### Subgroup analysis of viral load suppression rate

The subgroup analysis demonstrated that the rate of viral load suppression varied significantly across different populations and regions of Ethiopia. Children were found to have a higher rate of viral load suppression (83.1%) compared with adults (73.1%) and the general population (78.9%). Regionally, Tigray and Addis Ababa regions showed the highest rates of viral load suppression at 89.6% and 85.6% respectively, in contrast to Amhara and Oromia regions, which demonstrated the lowest rates of viral load suppression at 77.0% and 74.1% respectively. Despite these regional variations, all the regions were considered highly heterogeneous. When the studies were divided by sample size, studies with fewer than 500 participants had a viral load suppression rate of 77.1% while those with 500 or more participants had a rate of 79.3%. Overall, the rate of viral load suppression among individuals taking first-line ART was 76.6% (95% CI: 70.4%–81.8%). In contrast, those on mixed regimens had a viral load suppression rate of 83.6% (95% CI: 57.8%–95.0%), a rate that was imprecisely estimated due to the low number of studies included in the mixed regimens analysis. Finally, those on second and third-line ART had a viral load suppression rate of 78.1% (95% CI: 72.0%–83.2%) (Table 2).

**Table 2:** Subgroup analysis of pooled viral load suppression among people living with HIV in Ethiopia.

Variables	Category	Number of studies	Pooled Viral Load Suppression (%)	Heterogeneity within the studies	
				I2 (%)	p-value**
Population	General	14	78.9(68.0,86.8)	98.7	0.000
	Adults	18	73.1(65.1,79.8)	97.2	0.000
	Children	5	83.1(72.8,90.0)	91.3	0.000
Region	Addis Ababa	4	85.5(61.2,95.7)	94.6	0.000
	Amhara	21	77.0(70.4,82.5)	97.3	0.000
	Oromia	6	74.1 (57.4,85.9)	97.4	0.000
	Tigray	2	89.6(49.6,98.7)	79.4	0.000
Sample size	<500	32	77.1(71.4,81.9)	97.3	0.000
	≥500	7	79.3(62.5,89.8)	99.1	0.000
ART regimen	First-line ART	29	76.6(70.4,81.8)	97.8	0.000
	Mixed regimens	3	83.6 (57.8,95.0)	99.5	0.000
	Second or/and third-line ART	7	78.1 (72.0,83.2).	90.5	0.000

### Meta-regression analysis

A random-effects meta-regression using restricted maximum likelihood (REML) was performed to assess whether year of publication explained the observed heterogeneity. The analysis showed that year of publication was not a statistically significant predictor of the outcome (Coefficient = 0.060, p = 0.423), indicating no evidence of a temporal trend across studies. The model explained none of the between-study variability (R^2^ = 0.00%), and substantial residual heterogeneity remained (τ^2^ = 0.669; I^2^ = 97.91%). These findings suggest that the observed variation in effect sizes is largely attributable to factors other than publication year. (S1b Fig).

### Publication bias

Visual inspection of the funnel plot suggested asymmetry, indicating possible small-study effects (S2b Fig). This was supported by Egger’s regression test (p < 0.001), and Begg’s rank correlation test (p < 0.001). The trim-and-fill analysis suggested potential publication bias, imputing three missing studies on the left side of the funnel plot (S3b Fig). After adjustment, the pooled proportion decreased from 77.5 (95% CI: 72.6%–81.8%) to 76.2% (95% CI: 71.0–80.7%) under the random-effects model, suggesting a minimal change in the pooled estimate after adjustment for publication bias.

### Sensitivity analysis

Leave-one-out sensitivity analysis showed that the pooled viral load suppression estimate remained stable, ranging from 76.8% to 78.2%, compared with the overall estimate of 77.5% (95% CI: 72.5%–81.8%), indicating that no single study materially influenced the results (S4b Fig). Restricting the analysis to studies of participants receiving first-line ART yielded a pooled estimate of 76.6% (95% CI: 70.1%–82.1%), which was consistent with the primary analysis.

### Pooled effect of ART adherence on viral load suppression

**Fig 4:**
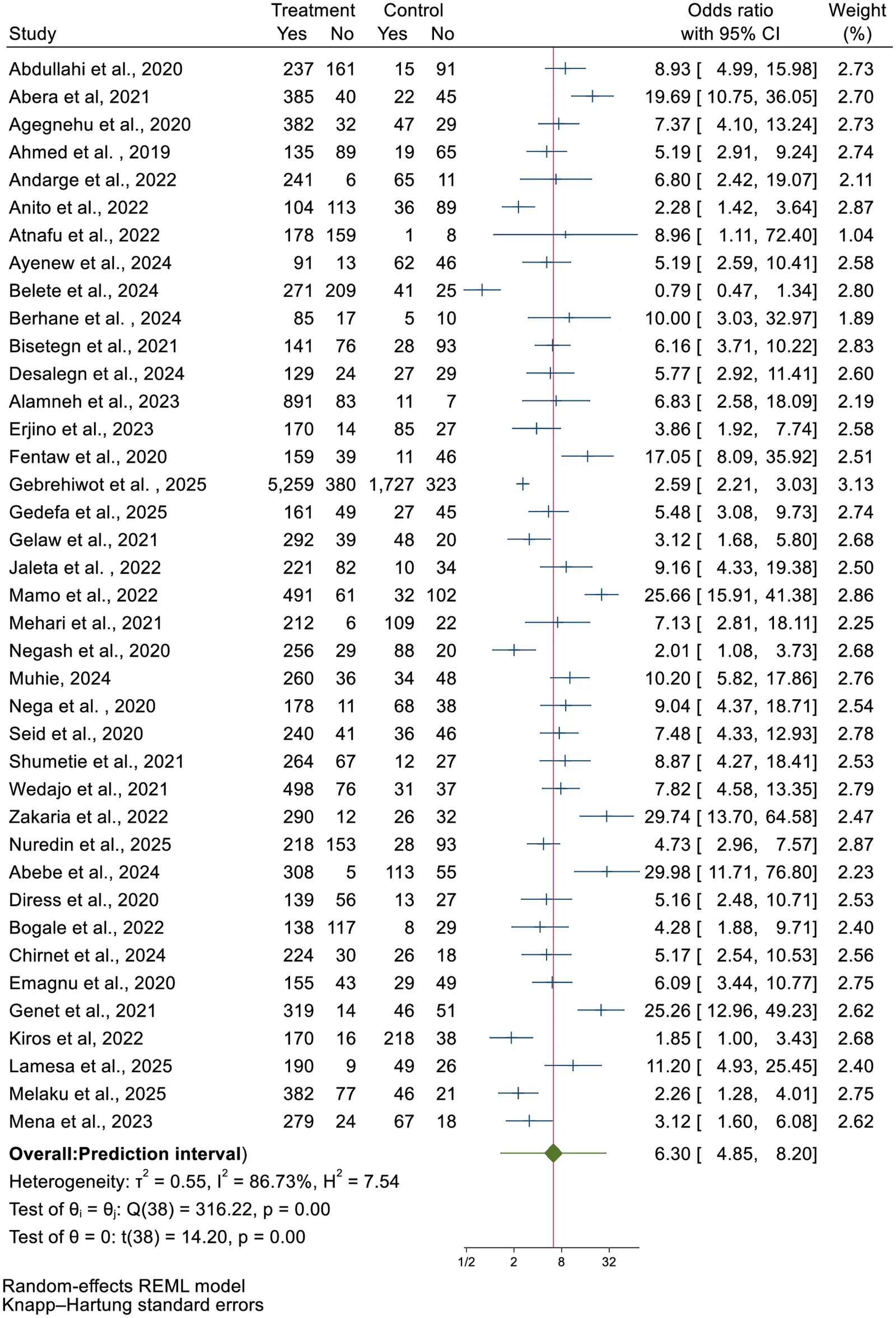
Forest plot showing the pooled odds ratio of the association between ART adherence and viral load suppression among people living with HIV in Ethiopia under a random-effects model.

A random-effects meta-analysis using restricted maximum likelihood (REML) with Knapp–Hartung adjustment was conducted to estimate the association between antiretroviral therapy (ART) adherence and viral load suppression among people living with HIV in Ethiopia. The pooled result showed that good ART adherence was significantly associated with higher odds of viral load suppression (pooled OR:6.30 (95% CI: 4.84–8.19). There was substantial heterogeneity among the included studies (I^2^ = 86.7%, τ^2^ = 0.55) (Fig 4).

### Meta-regression analysis

A random-effects meta-regression analysis using restricted maximum likelihood (REML) was performed to assess whether publication year explained the between-study heterogeneity in the association between antiretroviral therapy adherence and viral load suppression. The results indicated that publication year was not a statistically significant predictor of the effect size (coefficient = −0.092, p = 0.203), suggesting no evidence of a temporal trend in the strength of the association (S1c Fig). The model explained a small proportion of between-study variability (R^2^ = 3.05%), while substantial residual heterogeneity remained (τ^2^ = 0.535; I^2^ = 85.08%). These findings suggest that the variability in effect sizes across studies is likely driven by other factors not captured in the model.

### L’Abbé plot analysis

**Fig 5:**
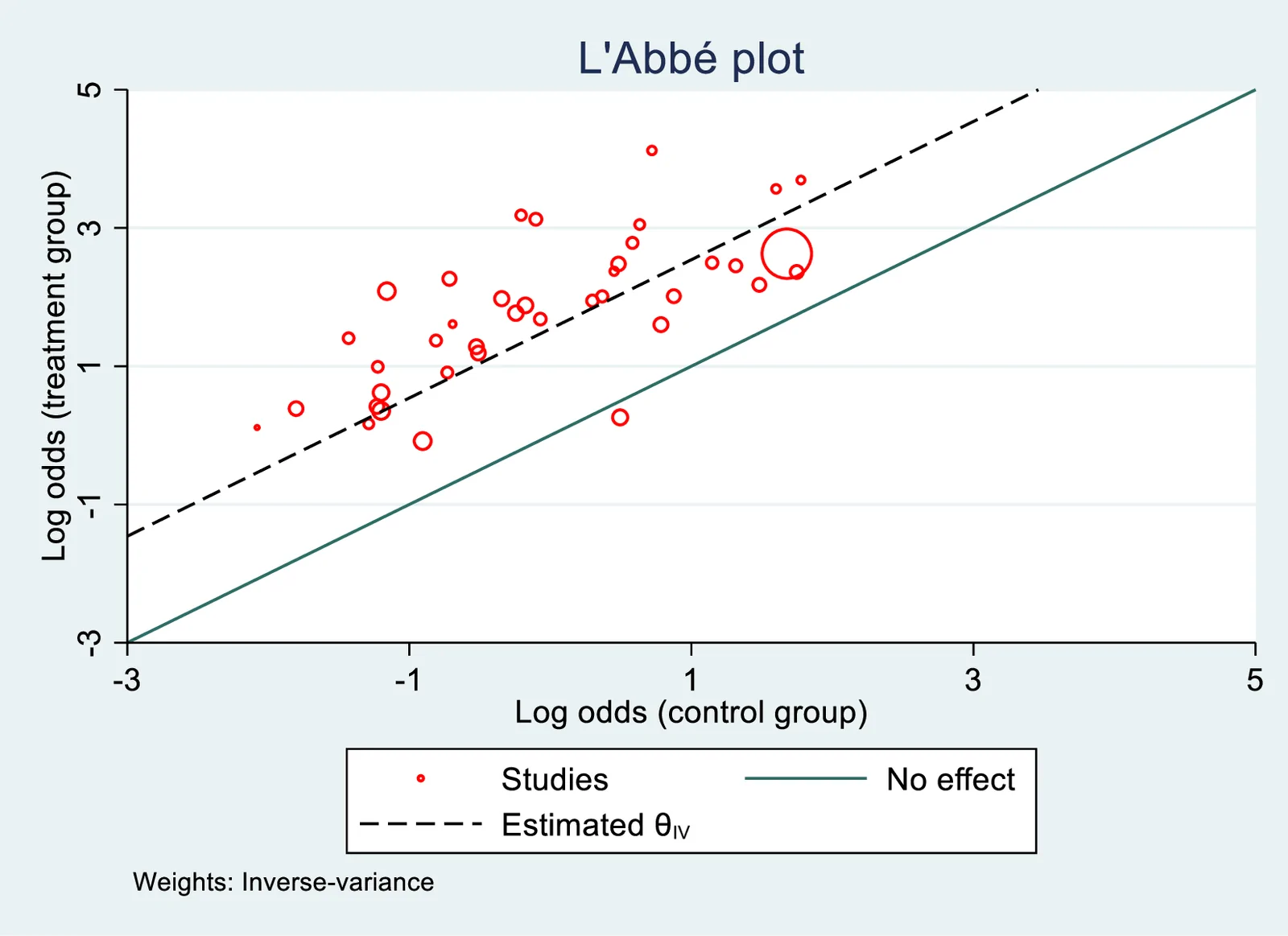
L’Abbé plot showing the relationship between ART adherence and viral load suppression across included studies, with each point representing a study and plotted proportions compared under a random-effects model.

The L’Abbé plot was used to visually assess the relationship between event rates in the exposed (good ART adherence) and control (poor adherence) groups across the included studies. Most studies were distributed above the line of no effect, indicating higher viral load suppression rates among individuals with good adherence compared to those with poor adherence. However, the studies were widely scattered around the pooled effect line, suggesting substantial between-study heterogeneity, which is consistent with the high I^2^ observed in the meta-analysis. Despite this variability, the overall trend remained above the line of no effect, indicating a consistent positive association between ART adherence and viral load suppression across studies. A small number of studies contributed more prominently due to larger weights, but no clear outliers with disproportionate influence were identified (Fig 5).

### Publication bias

Publication bias and small-study effects were assessed using funnel plot inspection and regression-based tests (Egger’s, Harbord, Peters) alongside Begg’s test under a random-effects REML model. The funnel plot appeared approximately symmetric, with no clear visual evidence of asymmetry (S2c Fig). Statistical tests did not indicate significant small-study effects: Egger test (p = 0.0682), Peters test (p = 0.537), and Begg’s test (p = 0.16) were all non-significant. However, Harbord’s test indicated borderline evidence of asymmetry (p = 0.035). To further explore the potential impact of publication bias, a nonparametric trim-and-fill analysis using a random-effects (REML) model was performed. The method imputed 11 potentially missing studies on the left side of the funnel plot. The observed pooled effect was 6.30 (95% CI: 4.84–8.19), which decreased to 4.40 (95% CI: 3.31–5.85) after adjustment. Although the adjusted estimate was lower, the association remained statistically significant, indicating that publication bias may have slightly inflated the pooled effect size but did not fully account for the observed association (S3c Fig),

### Sensitivity analysis

Leave-one-out sensitivity analysis showed that the pooled odds ratio remained stable, ranging from 6.03 to 6.66 compared with the overall estimate of 6.30 (95% CI: 4.84–8.19), indicating that no individual study substantially influenced the pooled effect (S4c Fig).

## Discussion

This systematic review and meta-analysis synthesized evidence from 39 studies conducted in Ethiopia between 2018 and 2025 to examine both the level of antiretroviral therapy (ART) adherence and its effect on viral load suppression among people living with HIV. The findings indicate that the pooled prevalence of viral load suppression was 77.5%, while the pooled prevalence of good ART adherence was 79.4%. In addition, good adherence was strongly associated with viral load suppression, with a pooled odds ratio of 6.30 (95% CI: 4.84–8.19). These findings highlight both progresses made through ART scale-up efforts in Ethiopia as well as persistent gaps relative to global targets. Moreover, it emphasizes ART adherence as a critical determinant of virological outcomes in HIV care.

The pooled prevalence of good ART adherence in Ethiopia was 79.4%, which is also below the optimal threshold (>95%) set by UNAIDS 95-95-95 targets for 2030 [7]. Our finding was comparable to other African settings where adherence ranges from 43–84%, with considerable regional variation. A slightly higher rate of good ART adherence was observed in our study than in reports from East African countries (69%–73%). Potential barriers to ART adherence are multifaceted, spanning individual-level issues such as mental health issues, lack of motivation, and substance abuse; social barriers including stigma and intimate partner violence; economic factors, including food insecurity, transport costs, and income instability; structural and programmatic barriers such as resource allocation, accessibility of health care settings, and service interruptions due to factors like war, and COVID-19 pandemic [15,17,18,67–69]. In subgroup analysis, adherence levels were comparable across Oromia, Amhara, and Addis Ababa regions, while adults showed a slightly higher ART adherence than children (80.7% vs 78.9%).

In the current review, the pooled viral load suppression rate in Ethiopia was 77.5%. Compared with the Joint United Nations Programme on HIV/AIDS (UNAIDS) 95-95-95 targets of the Fast-Track strategy to end the AIDS epidemic by 2030, Ethiopia’s pooled viral load suppression rate falls short of both the interim target (90%) for 2020 and the end goal (95%) for 2030 [7]. This gap suggests that a substantial proportion of people living with HIV in Ethiopia are still not achieving optimal treatment outcomes. This pattern mirrors findings from other low- and middle-income countries (LMICs), where similar lower rates of viral load suppression were reported from Sub-Saharan Africa (80%), Nigeria (64%), and Ghana (79%) [70–72].

Potential reasons for the observed gap in virologic suppression from the global targets include healthcare infrastructure constraints, socioeconomic barriers, regional instability, HIV drug resistance, comorbidities such as tuberculosis or hepatitis B, and variations in programmatic implementation [14,73–77]. In LMICs such as Ethiopia, resource limitations may have constrained the scale and effectiveness of HIV program implementation, contributing to slower progress toward global targets. More recently, reductions in global HIV funding may have further strained HIV service delivery, while disruptions related to the coronavirus disease 2019 (COVID-19) pandemic also adversely affected access to HIV care and treatment [74–77].

Subgroup analyses revealed marked heterogeneity in virologic suppression rates across Ethiopian regions: Addis Ababa (85.5%) showed a relatively higher virologic suppression rate than Amhara (77%) and Oromia (74.1%). Such heterogeneity may be attributed to differences in healthcare access between urban/rural areas; regional disparities in resources or program quality; sociocultural factors affecting support; or population-specific barriers such as stigma among key groups like prisoners or adolescents [9,22,73,78]. In addition, children achieved higher viral load suppression rates than adults, contrary to recent systematic reviews reporting comparable suppression rates across age groups [12,79]. This difference may reflect socioeconomic and clinical factors, including ART regimen and treatment duration, as well as programmatic improvements in ART services targeting key populations, particularly children.

Although first-line ART regimens generally achieve better outcomes at the program level from previous evidence, our subgroup analysis revealed a higher pooled rate of virologic suppression and adherence among patients receiving second- or third-line ART regimens than among those receiving first-line regimens [80]. Similarly, a previous systematic review from Ethiopia reported a higher rate of virologic suppression among patients receiving second-line ART than among those receiving first-line ART, whereas a more recent review reported comparable rates of virologic suppression [12,79]. These findings likely reflect differences in study composition and post-failure care rather than true superiority of second- or third-line regimens. Several studies suggest that adherence may improve after switching regimens because patients with first-line treatment failure often receive intensified adherence counseling, closer viral load monitoring, and regimen optimization, which may contribute to higher suppression rates in selected second-line cohorts [81,82]. Therefore, this observed association should be interpreted carefully as a context-dependent pooled effect rather than evidence of superior efficacy of second- or third-line regimens.

Consistent with global evidence, including large meta-analyses, the present review found that good ART adherence was strongly associated with viral load suppression among Ethiopians living with HIV (pooled OR: 6.30, 95% CI: 4.84–8.19). This finding is consistent with established biological and clinical understanding that sustained and consistent intake of ART maintains adequate drug plasma concentrations, thereby inhibiting viral replication and allowing suppression of HIV RNA levels. This relationship holds across diverse settings: studies show that even modest reductions in adherence (<90%) can significantly increase risk of virologic failure/resistance development [18,24].

The observed association aligns with global HIV treatment targets emphasized by UNAIDS, which aims for a high proportion of individuals on ART to achieve and maintain viral suppression as part of the 95–95–95 targets [7]. Adherence is a critical step in this cascade, as consistent ART use is one of the major factors to achieve viral suppression. The findings from this meta-analysis therefore underscore the importance of adherence-focused interventions in improving treatment outcomes such as virologic suppression.

### Implications

These findings underscore the need for multifaceted interventions addressing both individual-level barriers (e.g., mental health support, counseling) and structural determinants (e.g., poverty alleviation, food security programs). Strengthening ART adherence through targeted interventions such as patient education, counseling, adherence reminders, community-based support systems, and differentiated service delivery models can significantly improve viral suppression rates. Integrating adherence support into HIV care services is essential to achieving optimal treatment outcomes.

Furthermore, the findings suggest that adherence should remain a key focus in national HIV programs in Ethiopia. Enhancing adherence not only improves individual patient outcomes but also contributes to broader public health goals by reducing viral load at the population level, thereby lowering the risk of HIV transmission. This aligns with the treatment-as-prevention strategy, which relies on sustained viral suppression to curb the HIV epidemic.

### Limitations

This study has several limitations. All included studies were observational, predominantly cross-sectional, which limits causal inference and may leave residual confounding unaddressed. Viral load suppression was standardized using the WHO definition (<1000 copies/mL), excluding studies using lower thresholds (e.g., < 50 or 200 copies/mL), which may have reduced the number of eligible studies and introduced selection bias. Substantial heterogeneity was observed, likely due to differences in study populations, settings, ART programs, and measurement approaches. The timing of ART adherence assessment relative to viral load measurement was inconsistently reported across studies, which may have contributed to heterogeneity. Additionally, limited reporting of ART regimen-specific outcomes prevented assessment of the effect of regimen transitions, including the shift toward dolutegravir-based regimens, on adherence and viral suppression. Regional and subgroup analyses should also be interpreted cautiously due to uneven study representation and limited evidence in some categories. Although publication bias assessments showed no consistent evidence of bias, selective reporting and underrepresentation of certain populations cannot be excluded. Despite these limitations, this study provides comprehensive evidence on the association between ART adherence and viral suppression among people living with HIV in Ethiopia.

## Conclusion

This systematic review and meta-analysis demonstrate that ART adherence and viral load suppression among people living with HIV in Ethiopia are relatively high but remain below global targets. The pooled estimates indicate that approximately four out of five individuals achieve good adherence and viral suppression. Importantly, good ART adherence was strongly associated with viral load suppression.

These findings underscore ART adherence as a key modifiable determinant of treatment outcomes and highlight the need for sustained and context-specific interventions to improve adherence levels. Overall, efforts to scale up adherence-promoting strategies should remain a central component of HIV care programs in Ethiopia to achieve durable viral suppression and long-term treatment success.

## Supporting information

S1 File A PRISMA checklist for this systematic review and meta-analysis. (DOCX)

S2 File Search strategy for this systematic review and meta-analysis studies. (DOCX)

S3 File Quality assessment of selected studies. (XLSX)

S4 File Data extraction template for the effect of antiretroviral therapy adherence on viral load suppression rate among people living with HIV in Ethiopia. (DOCX)

S1 Fig Meta-regression analysis for the effect of antiretroviral therapy adherence on viral load suppression rate among people living with HIV in Ethiopia. (PDF)

S2 Fig Funnel plot publication bias for the effect of antiretroviral therapy adherence on viral load suppression rate among people living with HIV in Ethiopia. (PDF)

S3 Fig Trim-and-fill analysis for the effect of antiretroviral therapy adherence on viral load suppression rate among people living with HIV in Ethiopia. (PDF)

S4 Fig Leave-one-out sensitivity analysis for the effect of antiretroviral therapy adherence on viral load suppression rate among people living with HIV in Ethiopia. (PDF)

## Abbreviations

ART: Antiretroviral therapy
CI: Confidence Interval
SRS: Simple random sampling
OR: Odds Ratio
NOS: Newcastle–Ottawa Scale
HIV: Human Immunodeficiency Viruses
PRISMA: Preferred Reporting Items for Systematic Review and Meta-Analysis
PLHIV: People Living with Human Immunodeficiency Viruses
