# Supplementary material for: The effect of antiretroviral therapy adherence on viral load suppression rate among people living with HIV in Ethiopia: A systematic review and meta-analysis
Source: PLoS One. 2026 Sep 15;21(9):e0358352. doi: 10.1371/journal.pone.0358352 (PMC13577418; doi:10.1371/journal.pone.0358352)
Supplement: Supplementary file 1 [file pone.0358352.s005.pdf]

Meta regression on Adherence level

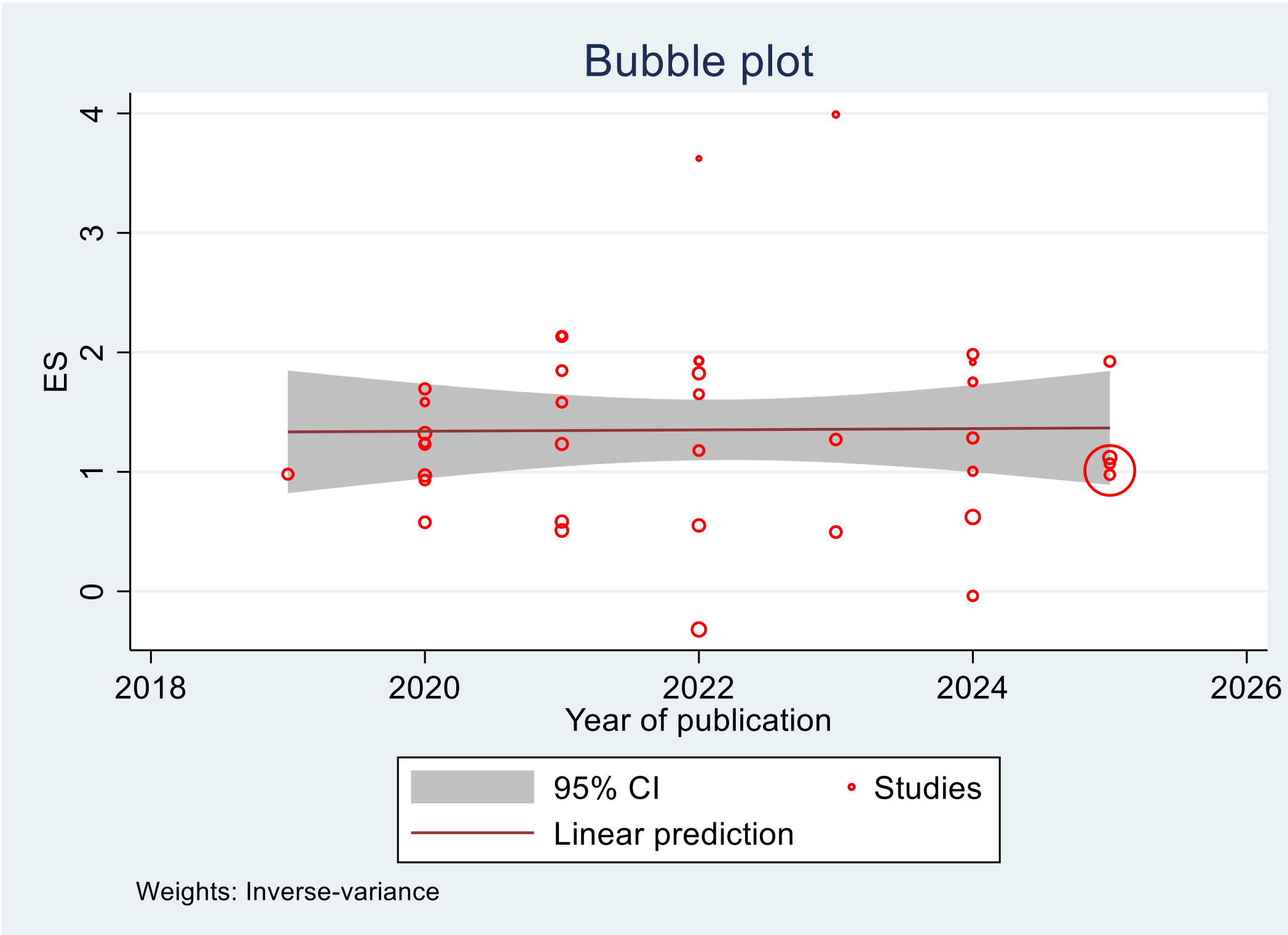

S1 Fig a: Bubble plot of meta-regression analysis showing the association between study year and adherence level, with bubble size proportional to study weight under a random-effects model.

Meta regression on Viral load

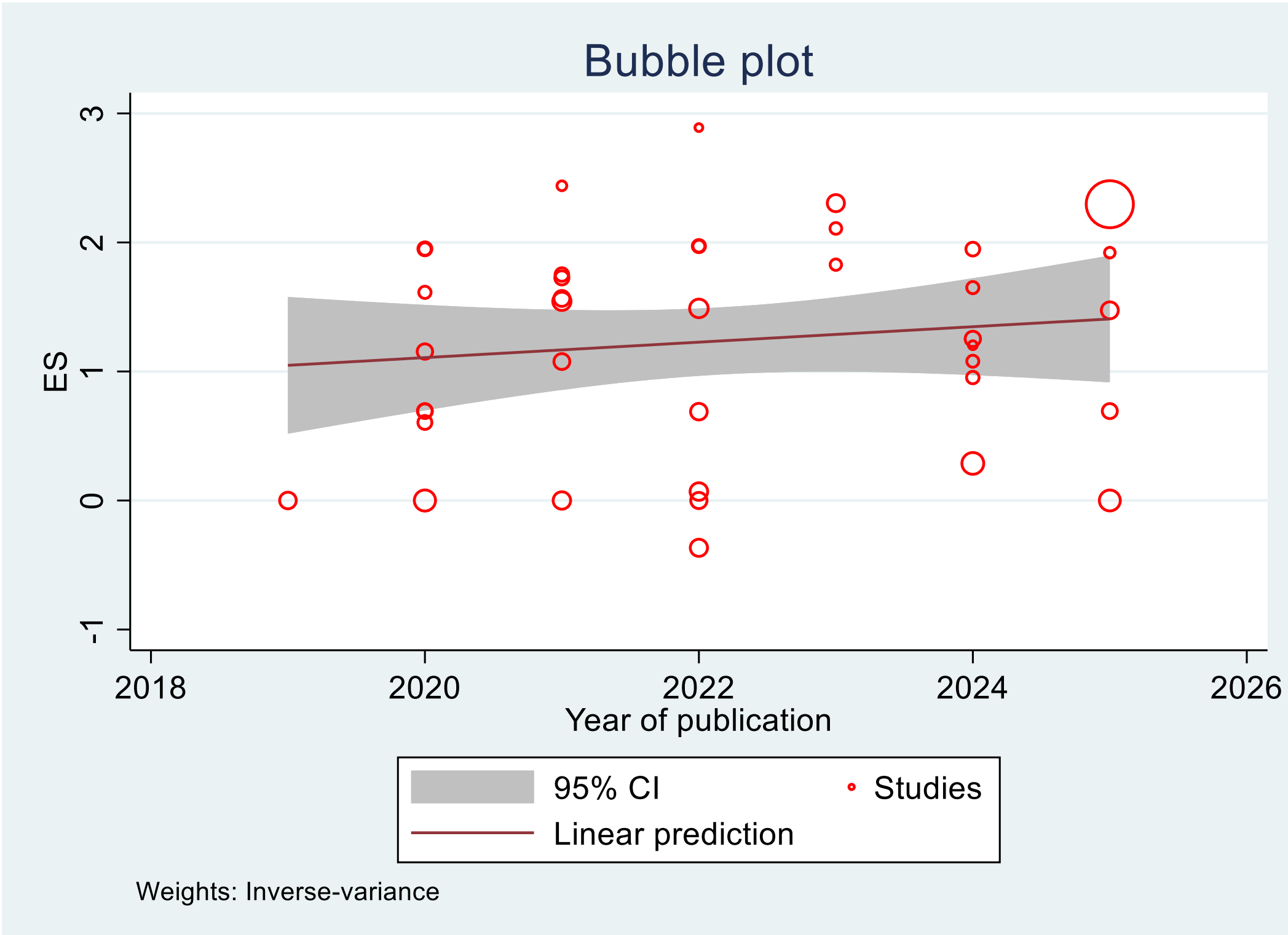

S1 Fig b: Bubble plot of meta-regression showing the association between study year and viral load suppression, with bubble size proportional to study weight under a random-effects model.

Meta regression the effect of adherence on load

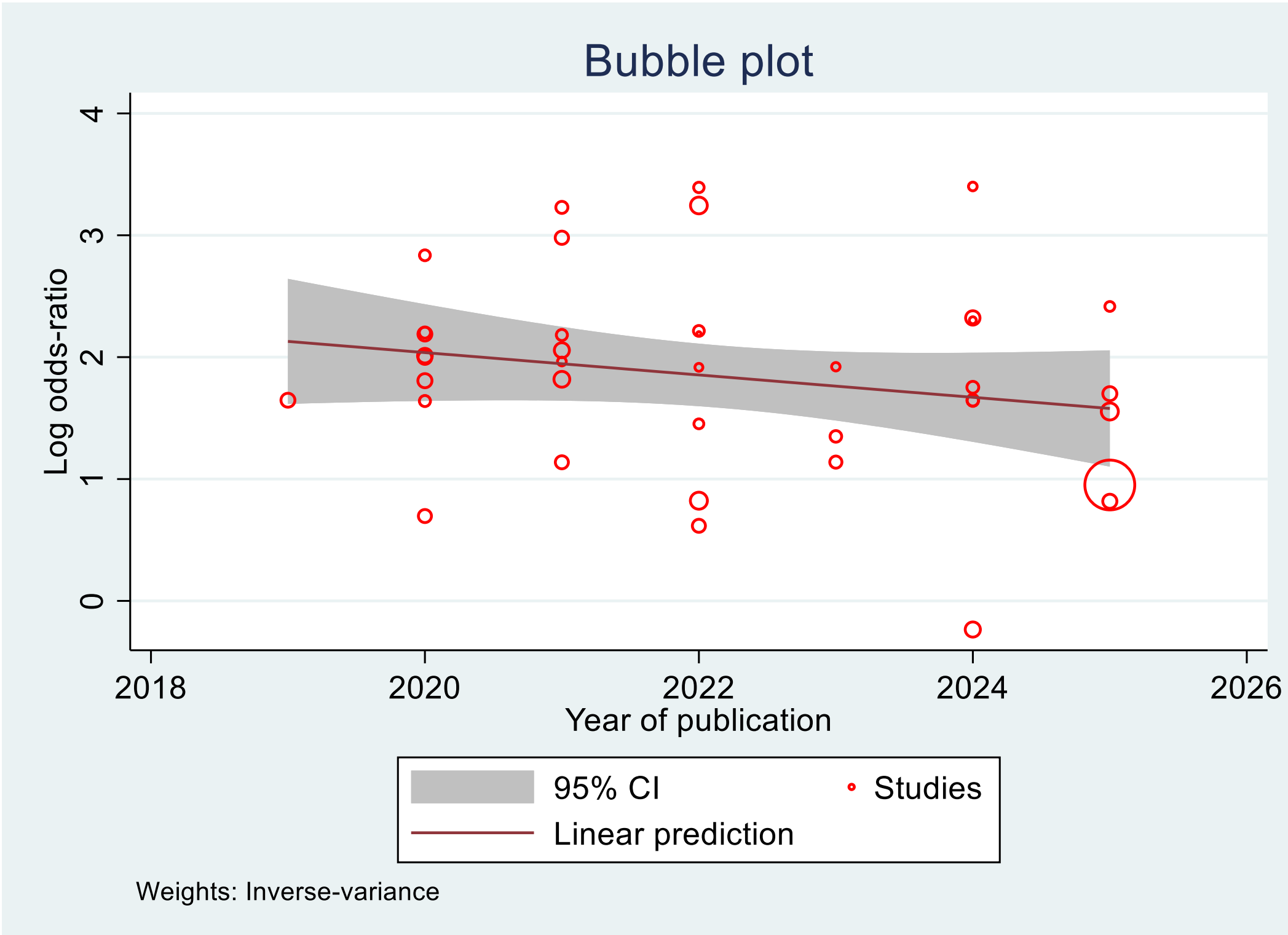

S1 Fig c: Bubble plot of meta-regression assessing study-level factors associated with the relationship between ART adherence and viral load suppression, with bubble size proportional to study weight under a random-effects model.
