# Supplementary material for: The effect of antiretroviral therapy adherence on viral load suppression rate among people living with HIV in Ethiopia: A systematic review and meta-analysis
Source: PLoS One. 2026 Sep 15;21(9):e0358352. doi: 10.1371/journal.pone.0358352 (PMC13577418; doi:10.1371/journal.pone.0358352)
Supplement: Supplementary file 2 [file pone.0358352.s006.pdf]

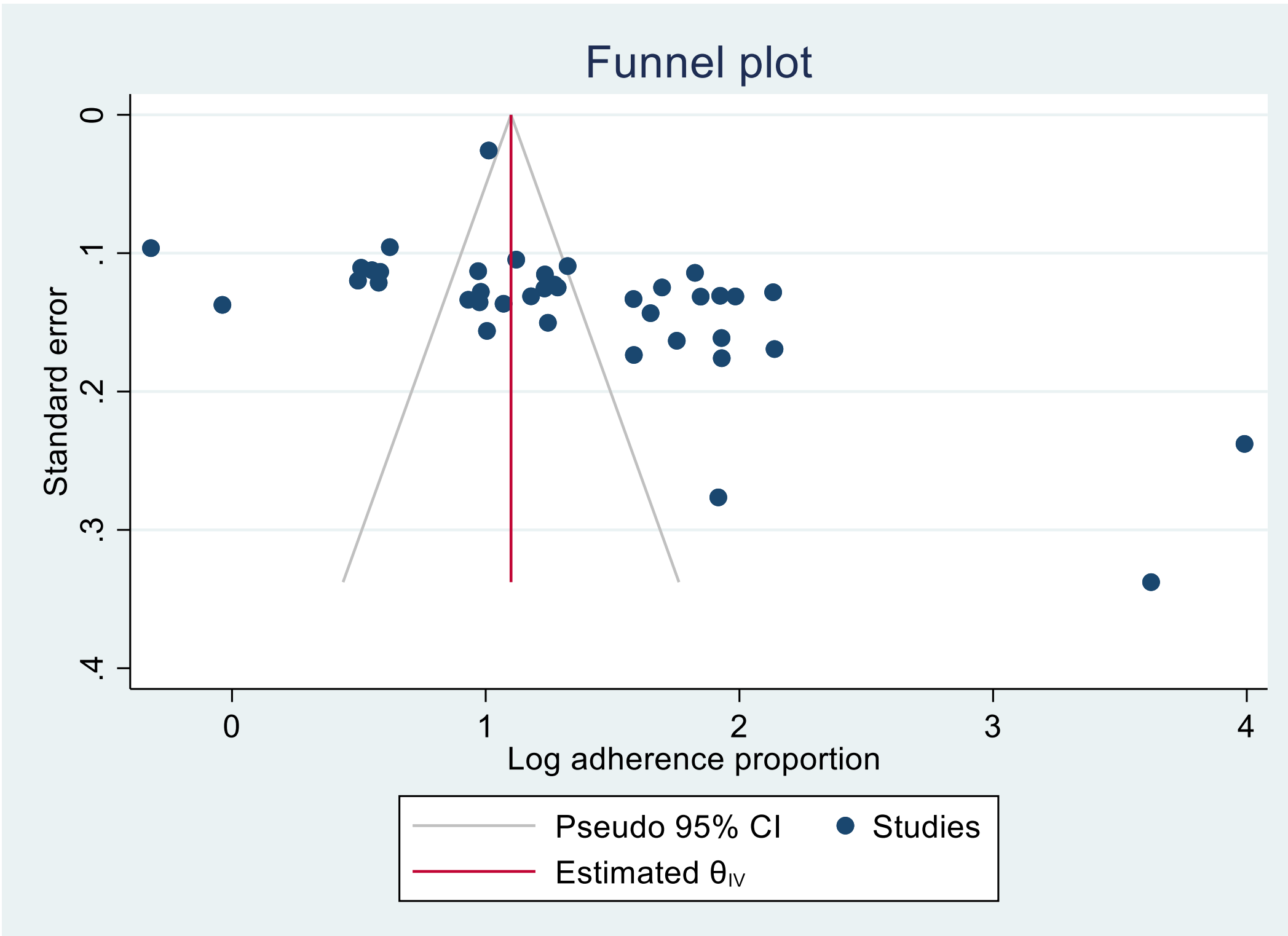

S2 Fig a:Funnel plot assessing publication bias for ART adherence estimates.

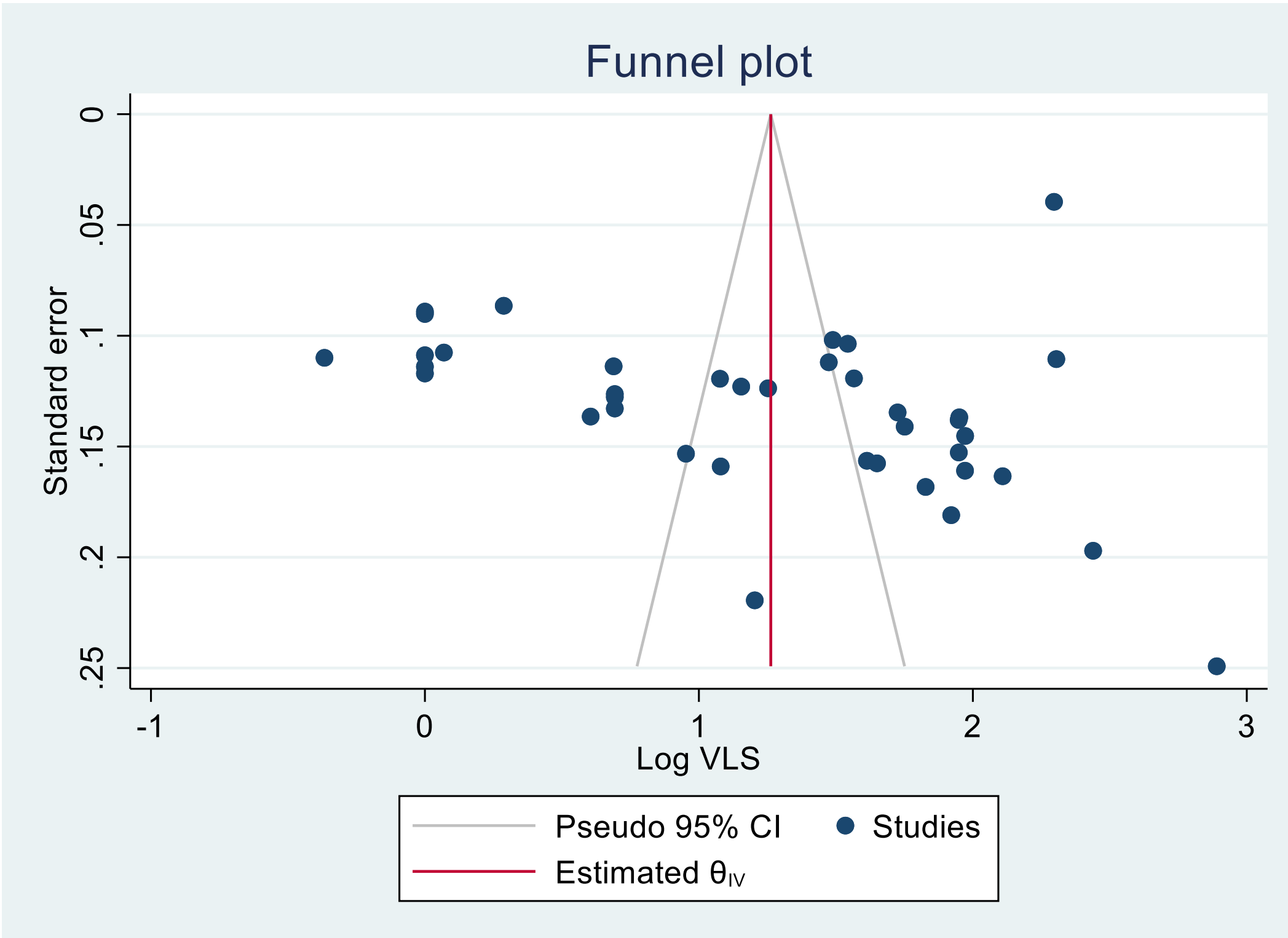

S2 Fig b: Funnel plot assessing publication bias for viral load suppression estimates.

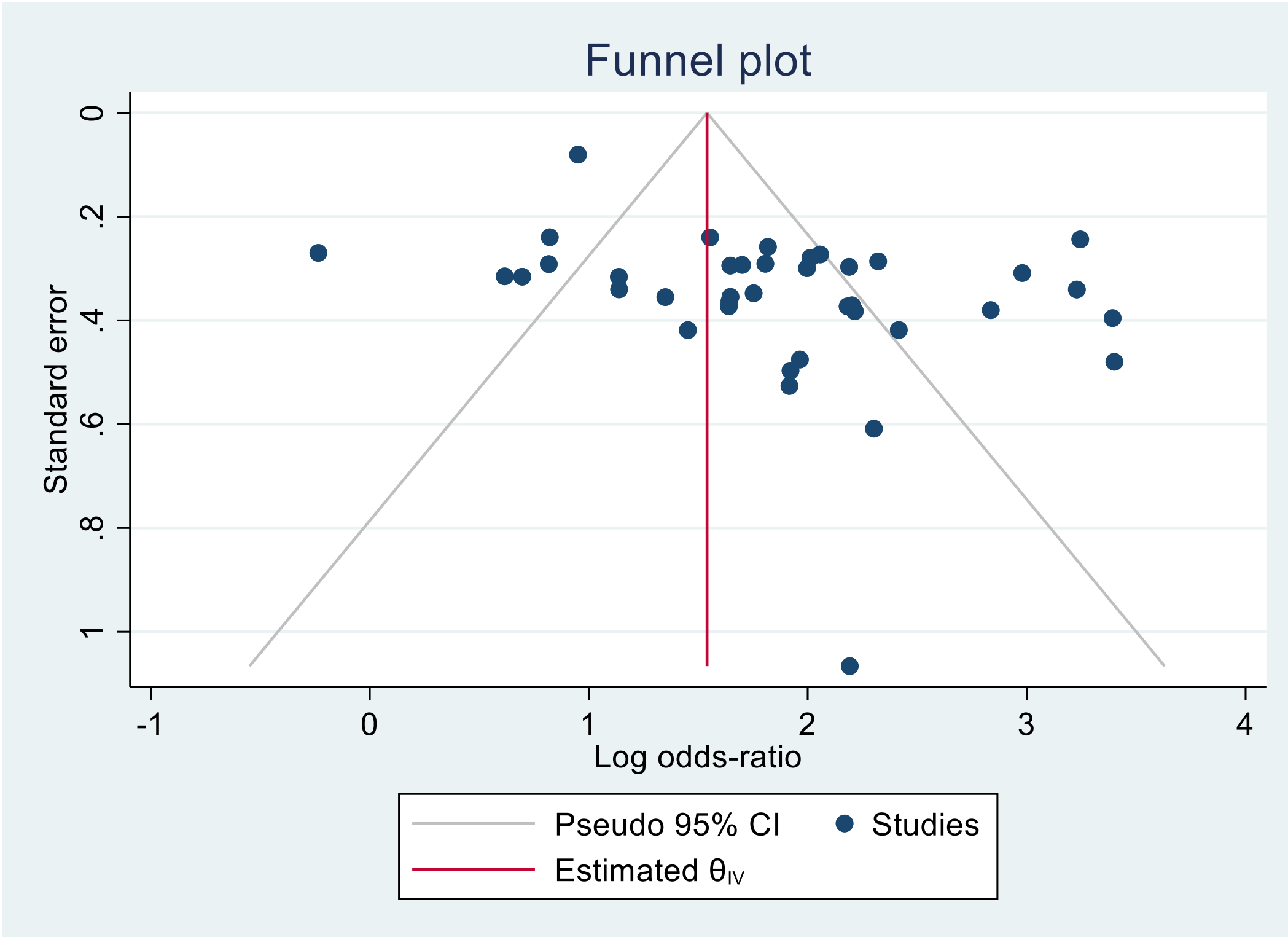

S2 Fig c: Funnel plot assessing publication bias for the association between ART adherence and viral load suppression.
