# Supplementary material for: The effect of antiretroviral therapy adherence on viral load suppression rate among people living with HIV in Ethiopia: A systematic review and meta-analysis
Source: PLoS One. 2026 Sep 15;21(9):e0358352. doi: 10.1371/journal.pone.0358352 (PMC13577418; doi:10.1371/journal.pone.0358352)
Supplement: Supplementary file 3 [file pone.0358352.s007.pdf]

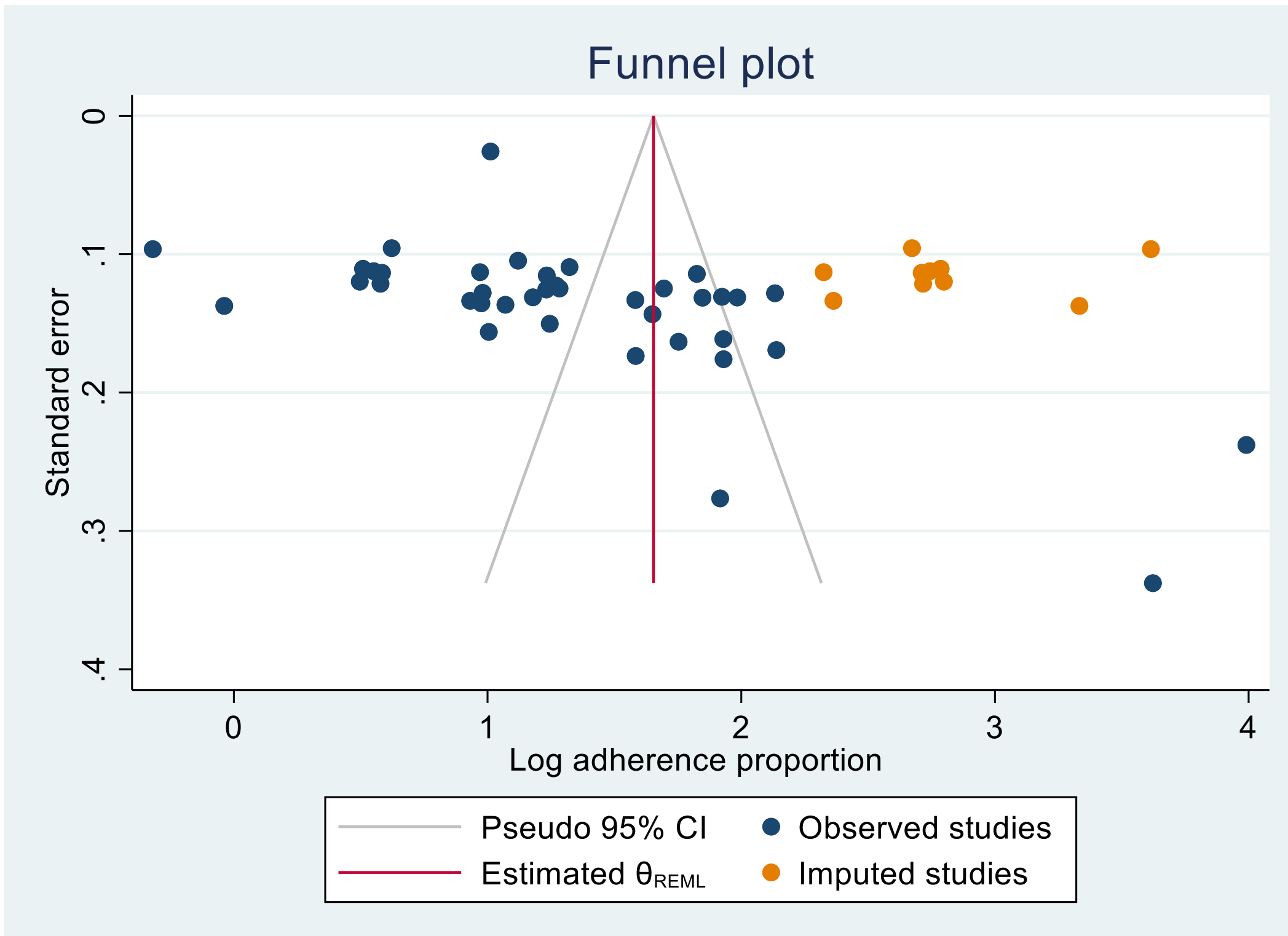

S3 Fig a:Trim-and-fill analysis of publication bias for ART adherence showing observed and imputed studies and the adjusted pooled proportion under a random-effects model.

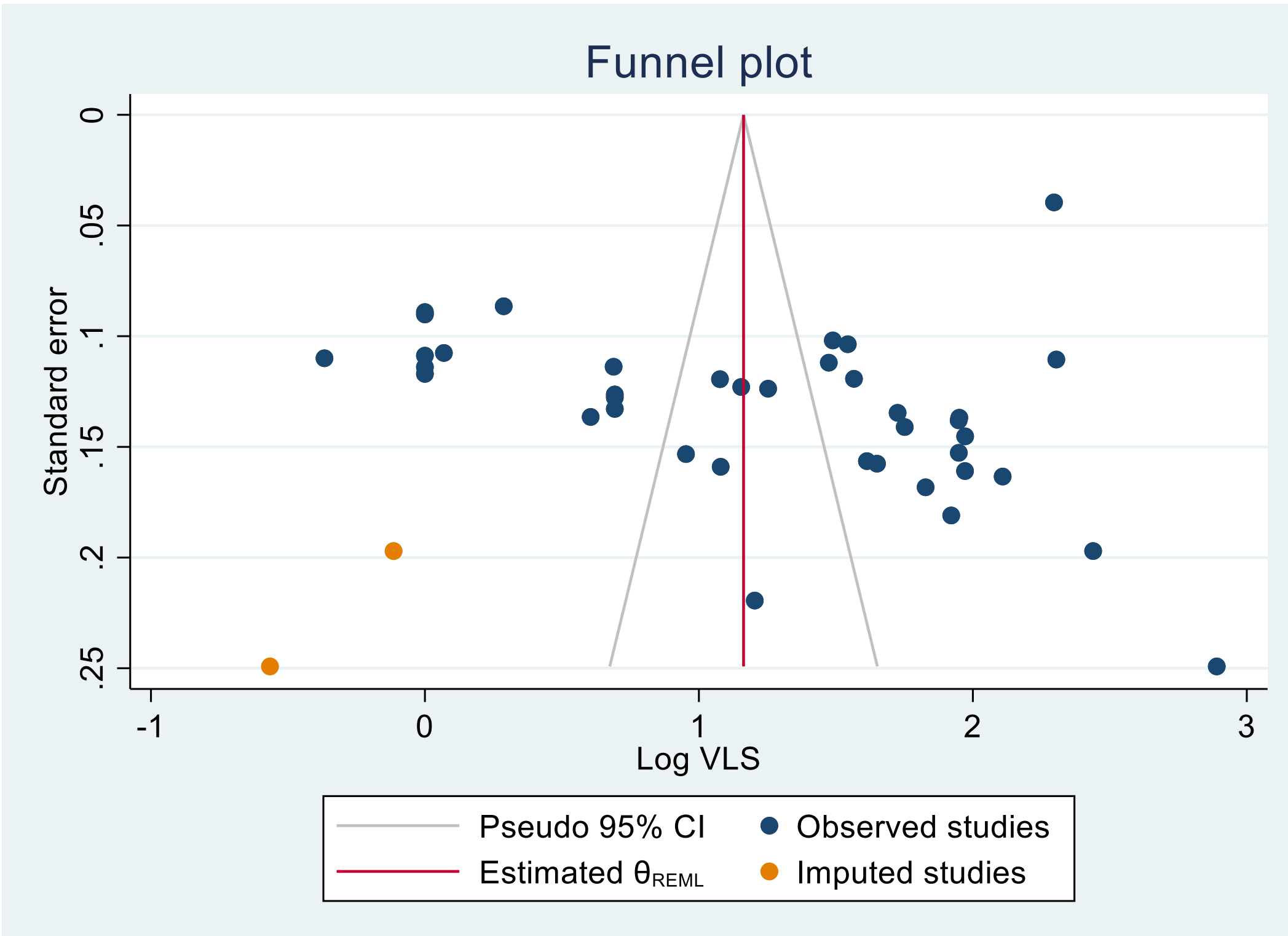

S3 Fig b: Trim-and-fill analysis of publication bias for viral load suppression showing observed and imputed studies and the adjusted pooled proportion under a random-effects model.

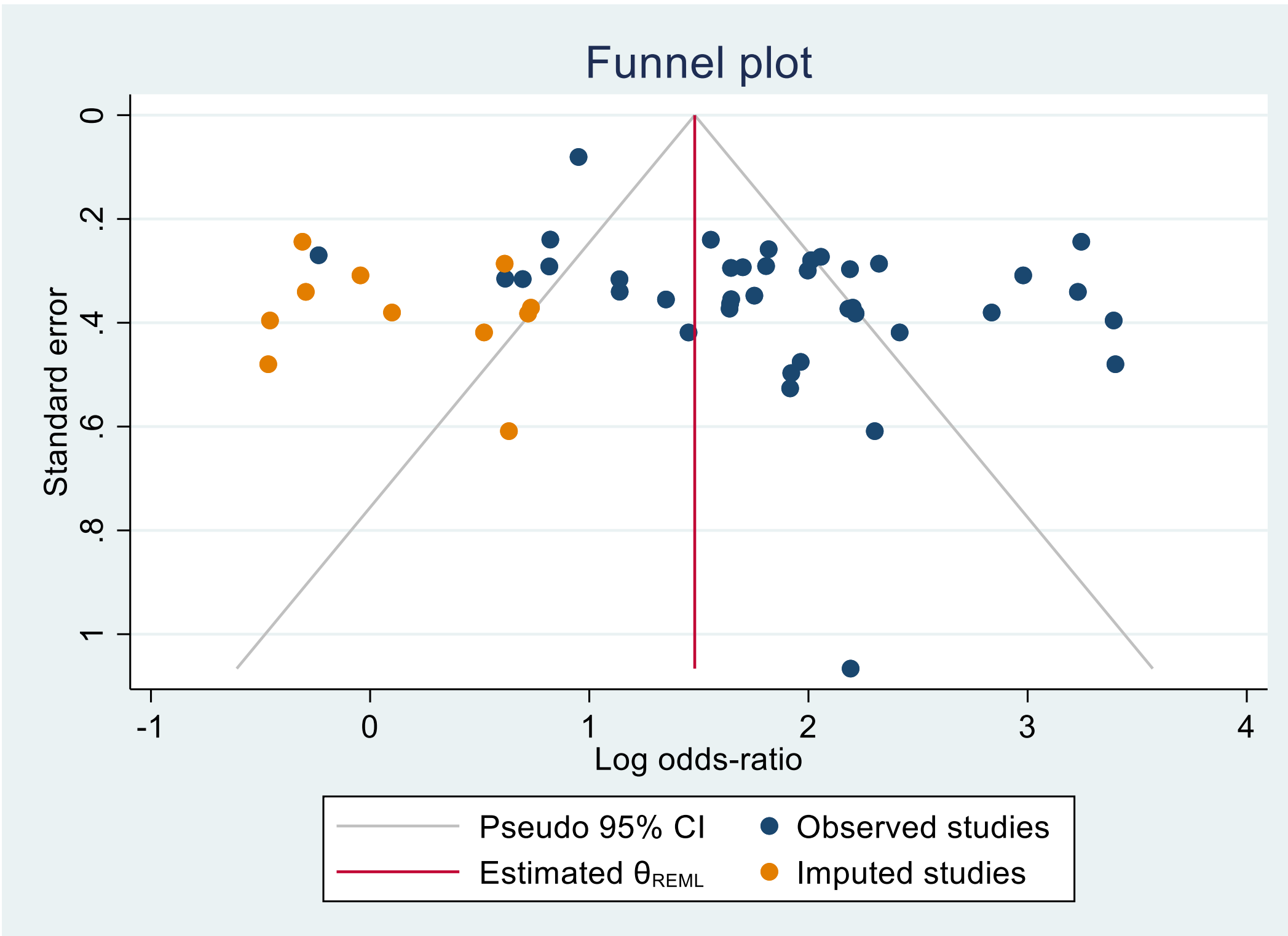

S3 Fig c:Trim-and-fill analysis of publication bias for the association between ART adherence and viral load suppression showing observed and imputed studies and the adjusted pooled odds ratio under a random-effects model.
