# Supplementary material for: The effect of antiretroviral therapy adherence on viral load suppression rate among people living with HIV in Ethiopia: A systematic review and meta-analysis
Source: PLoS One. 2026 Sep 15;21(9):e0358352. doi: 10.1371/journal.pone.0358352 (PMC13577418; doi:10.1371/journal.pone.0358352)
Supplement: Supplementary file 4 [file pone.0358352.s008.pdf]

Adherence level

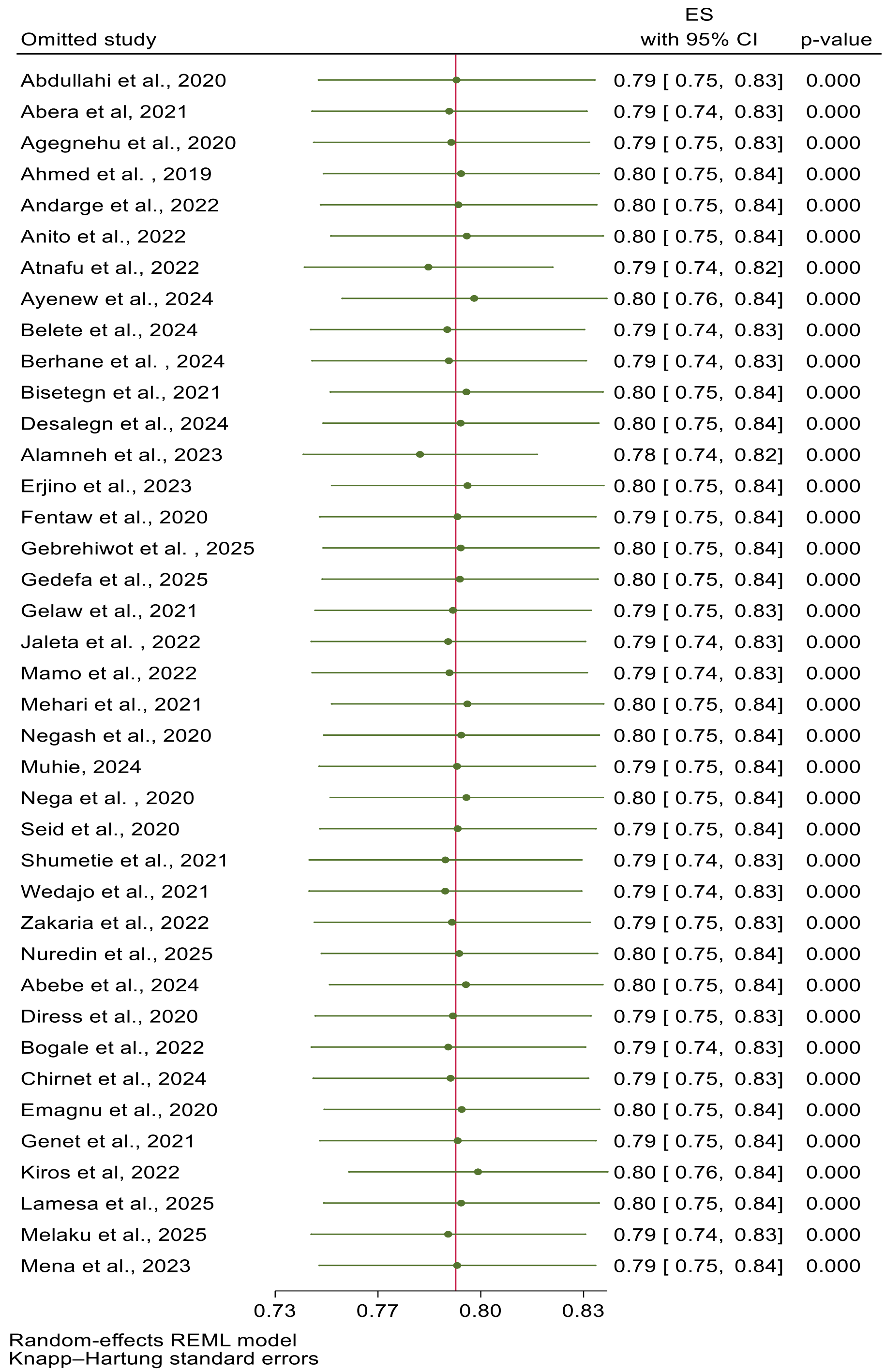

S4 Fig a:Leave-one-out sensitivity analysis for ART adherence showing the influence of individual studies on the pooled estimate.

Viral load suppression rate

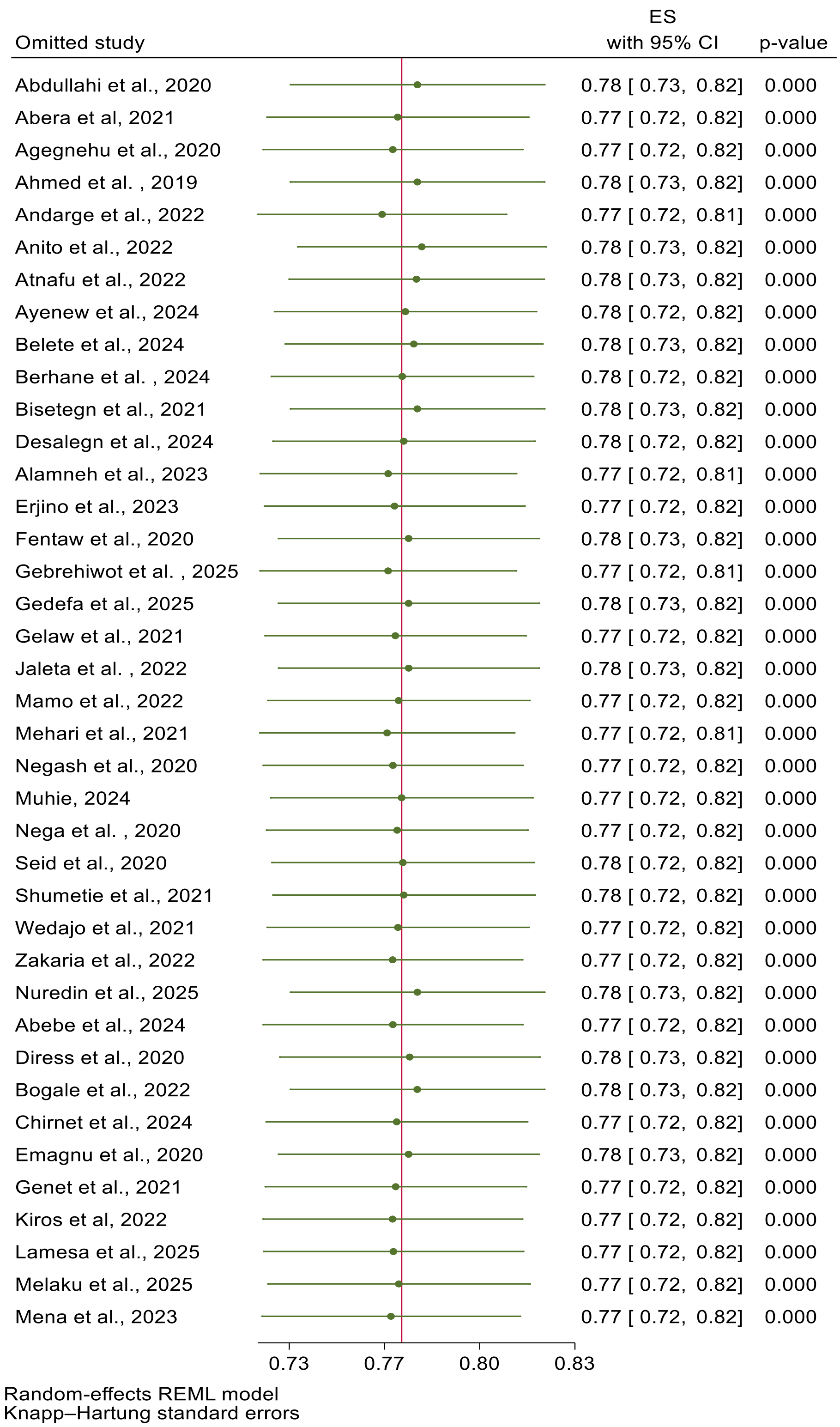

S4 Fig b: Leave-one-out sensitivity analysis for viral load suppression showing the influence of individual studies on the pooled estimate.

Sensitivity analysis of the effect of adherence on viral load suppression

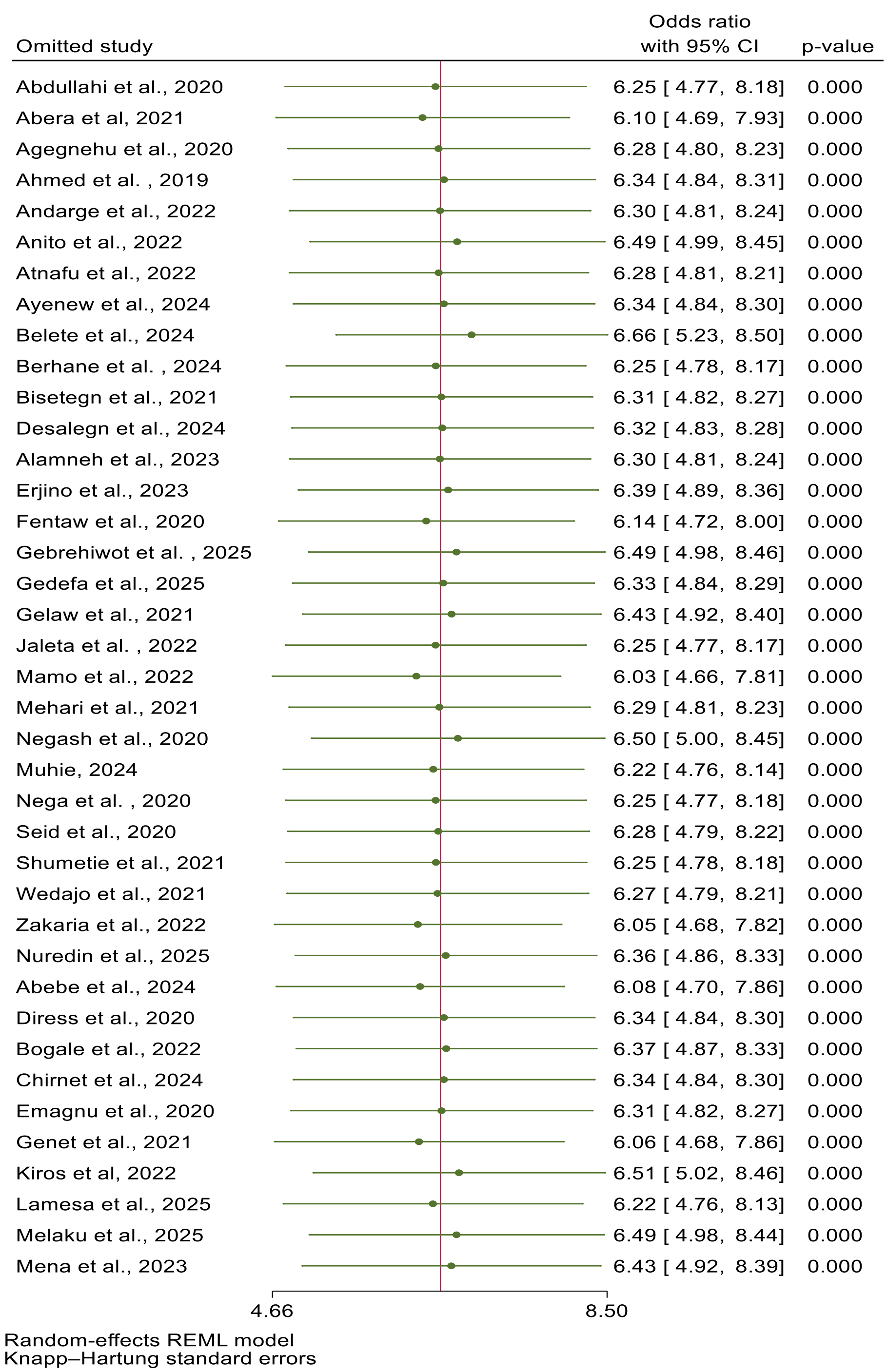

S4 Fig c: Leave-one-out sensitivity analysis for the association between ART adherence and viral load suppression showing the influence of individual studies on the pooled odds ratio.
