# Supplementary material for: The effect of antiretroviral therapy adherence on viral load suppression rate among people living with HIV in Ethiopia: A systematic review and meta-analysis
Source: PLoS One. 2026 Sep 15;21(9):e0358352. doi: 10.1371/journal.pone.0358352 (PMC13577418; doi:10.1371/journal.pone.0358352)
Supplement: Supplementary file 5 [file pone.0358352.s009.pdf]

Reviewer Comments

Title: **The effect of antiretroviral therapy adherence on viral load suppression rate among people living with HIV in Ethiopia: a systematic review and meta-analysis**

The authors have conducted an important study on a topic of significant public health relevance. The study is well designed and has considerable merit. The manuscript would be further strengthened by addressing the comments and suggestions outlined below.

Comments

1. Overall, the manuscript is well written and thoughtfully structured. However, several minor issues require attention before publication. These include inconsistent use of abbreviations after their initial definition, typographical errors (e.g., "art" instead of "ART"), and other minor editorial and formatting issues that should be carefully reviewed and corrected throughout the manuscript.
2. On page 5, the eligibility criteria define good ART adherence using multiple measurement approaches (e.g., pill counts, pharmacy refill records, self-report, and electronic monitoring). It is unclear whether all included studies used these methods or whether adherence measurement varied across studies. If variability existed, this should be explicitly described. Additionally, self-reported adherence is subject to recall and social desirability bias; therefore, it would be preferable to specify the actual adherence measurement methods used in the included studies rather than listing all possible approaches.
3. Did the included studies consider the timing of adherence assessment relative to viral load testing? Specifically, was adherence measured before, concurrent with, or after viral load assessment? Any variability in the timing of adherence measurement across studies should be clearly described, as it may affect the interpretation of the association between adherence and viral suppression.
4. WHO guidelines, adopted by Ethiopia and many other countries, recommend viral load testing at 6 months after ART initiation, at 12 months, and annually thereafter. Since viral load suppression may vary across these time points, it is important to

clarify whether the timing of viral load assessment was considered during study selection or analysis. If this factor was controlled for, it should be explicitly described in the methods section, as differences in testing intervals may contribute to heterogeneity across studies. At list this issue need to be acknowledged in the discussion.

5. On pages 10–11, the subgroup analysis indicates that:
   i. Children achieved higher viral load suppression than adults. This finding appears inconsistent with many published studies and routine program data, which generally report lower suppression among children. The authors should provide a stronger explanation for this observation and explore potential reasons
   ii. Given the substantial variation in the number of studies across regions, regional comparisons of VLS should be interpreted with caution. In particular, findings for Tigray, based on only two studies, may be unreliable and potentially misleading. Consider toning down the interpretation for Tigray and focusing on regions represented by at least three studies.
   iii. The findings related to regimen type are also inconsistent with existing evidence and therefore require stronger justification and clearer interpretation. The authors should provide a more detailed explanation for these results, considering possible factors such as differences in patient characteristics, prior treatment failure, adherence patterns, and the composition of the included studies.
6. Minor suggestion: Consider presenting the findings in the order of the HIV care continuum, consistent with the title of your review (i.e., adherence followed by viral load suppression throughout the MS).

7. In P2 under the discussion section:
   I. Access to viral load testing may not adequately explain the observed differences, as this review included treatment-experienced PLHIV who had

undergone viral load testing. This explanation is more applicable to population-level VLS studies than to ART cohort studies and should therefore be reconsidered.

II. In the same paragraph, the low VLS rate in Ethiopia is attributed to recent funding cuts and their impact on the HIV response. This interpretation requires stronger supporting evidence. Do studies conducted before the funding cuts support this argument? Moreover, gaps in viral load testing are not the focus of this review and do not adequately explain VLS among treatment-experienced PLHIV. Given that VLS in Ethiopia has generally improved over time and is approaching global targets, any explanation for the observed findings should be evidence-based and appropriately contextualized to strengthen the manuscript and its relevance for the national HIV program.

8. Regarding subgroup analysis (discussion section P3)
   I. As noted in Comment 5(ii), caution is warranted when interpreting findings from regions represented by only a few studies. The discussion should acknowledge this limitation and avoid drawing strong conclusions from subgroups with limited evidence..
   II. I did not find pregnant women included in the subgroup analysis, although they are discussed later in the manuscript. The supplementary table indicates that one study involving pregnant women was included. Please revisit this section to ensure consistency between the included studies, subgroup analysis, and discussion, or clarify why this study was not incorporated into the subgroup analysis.
9. Similar comments with #8 on subgroup analysis for adherence

Overall, the methodological rigor in study selection, risk of bias assessment, and statistical analysis is commendable. However, the manuscript would be strengthened by addressing the comments above to improve clarity, interpretation, and its ability to fully answer the primary review question.
