# Supplementary material for: The effect of antiretroviral therapy adherence on viral load suppression rate among people living with HIV in Ethiopia: A systematic review and meta-analysis
Source: PLoS One. 2026 Sep 15;21(9):e0358352. doi: 10.1371/journal.pone.0358352 (PMC13577418; doi:10.1371/journal.pone.0358352)
Supplement: Supplementary file 7 [file pone.0358352.s002.docx]

|  | Search | Query | Items found |
| --- | --- | --- | --- |
| PubMed | 1 | "antiretroviral therapy, highly active"[MeSH Terms] OR "antiretroviral therapy"[Title/Abstract] OR "ART"[Title/Abstract] OR "HAART"[Title/Abstract] OR "antiretroviral"[Title/Abstract] | 258,264 |
|  | 2 | "medication adherence"[MeSH Terms] OR ("adherance"[All Fields] OR "adhere"[All Fields] OR "adhered"[All Fields] OR "adherence"[All Fields] OR "adherences"[All Fields] OR "adherent"[All Fields] OR "adherents"[All Fields] OR "adherer"[All Fields] OR "adherers"[All Fields] OR "adheres"[All Fields] OR "adhering"[All Fields]) OR ("treatment adherence and compliance"[MeSH Terms] OR ("treatment"[All Fields] AND "adherence"[All Fields] AND "compliance"[All Fields]) OR "treatment adherence and compliance"[All Fields] OR ("treatment"[All Fields] AND "adherence"[All Fields]) OR "treatment adherence"[All Fields]) OR ("medication adherence"[MeSH Terms] OR ("medication"[All Fields] AND "adherence"[All Fields]) OR "medication adherence"[All Fields]) OR ("compliances"[All Fields] OR "patient compliance"[MeSH Terms] OR ("patient"[All Fields] AND "compliance"[All Fields]) OR "patient compliance"[All Fields] OR "compliance"[All Fields] OR "compliance"[MeSH Terms]) | 861,255 |
|  | 3 | "viral load"[MeSH Terms] OR ("viral load"[MeSH Terms] OR ("viral"[All Fields] AND "load"[All Fields]) OR "viral load"[All Fields]) OR (("virally"[All Fields] OR "virals"[All Fields] OR "virology"[MeSH Terms] OR "virology"[All Fields] OR "viral"[All Fields]) AND ("suppress"[All Fields] OR "suppressed"[All Fields] OR "suppresser"[All Fields] OR "suppresses"[All Fields] OR "suppressibility"[All Fields] OR "suppressible"[All Fields] OR "suppressing"[All Fields] OR "suppression"[All Fields] OR "suppressions"[All Fields] OR "suppressive"[All Fields] OR "suppressives"[All Fields])) OR (("viral load"[MeSH Terms] OR ("viral"[All Fields] AND "load"[All Fields]) OR "viral load"[All Fields]) AND ("suppress"[All Fields] OR "suppressed"[All Fields] OR "suppresser"[All Fields] OR "suppresses"[All Fields] OR "suppressibility"[All Fields] OR "suppressible"[All Fields] OR "suppressing"[All Fields] OR "suppression"[All Fields] OR "suppressions"[All Fields] OR "suppressive"[All Fields] OR "suppressives"[All Fields])) OR (("virologically"[All Fields] OR "virology"[MeSH Terms] OR "virology"[All Fields] OR "virologic"[All Fields] OR "virological"[All Fields]) AND ("suppress"[All Fields] OR "suppressed"[All Fields] OR "suppresser"[All Fields] OR "suppresses"[All Fields] OR "suppressibility"[All Fields] OR "suppressible"[All Fields] OR "suppressing"[All Fields] OR "suppression"[All Fields] OR "suppressions"[All Fields] OR "suppressive"[All Fields] OR "suppressives"[All Fields])) OR (("virologically"[All Fields] OR "virology"[MeSH Terms] OR "virology"[All Fields] OR "virologic"[All Fields] OR "virological"[All Fields]) AND ("failure"[All Fields] OR "failures"[All Fields])) | 124,514 |
|  | 4 | "hiv infections"[MeSH Terms] OR ("hiv"[MeSH Terms] OR "hiv"[All Fields]) OR (("people s"[All Fields] OR "peopled"[All Fields] OR "peopling"[All Fields] OR "persons"[MeSH Terms] OR "persons"[All Fields] OR "people"[All Fields] OR "peoples"[All Fields]) AND ("lived"[All Fields] OR "lives"[All Fields] OR "living"[All Fields] OR "livings"[All Fields]) AND ("hiv"[MeSH Terms] OR "hiv"[All Fields])) OR ("plhiv"[All Fields] OR "plhiv s"[All Fields] OR "plhivs"[All Fields]) | 489,886 |
|  | 5 | "ethiopia"[MeSH Terms] OR "ethiopia"[MeSH Terms] OR "ethiopia"[All Fields] OR "ethiopia s"[All Fields] OR "ethiopian people"[Supplementary Concept] OR "ethiopian people"[All Fields] OR "ethiopian"[All Fields] OR "ethiopians"[All Fields] | 55762 |
|  | 6 | #1 and #2 and #3 and # 4 and # 5AND (2015:2025[pdat]) | 172 |
| Scoups |  | TITLE-ABS-KEY ( ( "antiretroviral therapy" OR antiretroviral* OR ART OR HAART ) AND ( adherence OR compliance OR "treatment adherence" OR "medication adherence" ) AND ( "viral load" OR "viral suppression" OR "viral load suppression" OR "virologic suppression" OR "virologic failure" ) AND ( HIV OR "human immunodeficiency virus" OR "people living with HIV" OR PLHIV ) AND ( Ethiopia OR Ethiopian ) ) AND PUBYEAR > 2015 AND PUBYEAR < 2026 | 160 |
| Science Direct |  | antiretroviral AND adherence AND "viral load" AND HIV AND Ethiopia | 348 |
| Googel scholar |  | "antiretroviral therapy adherence" "viral load suppression" "HIV" "Ethiopia" | 601 |
| Epistemonikos |  | (title:((title:("Antiretroviral Therapy, Highly Active" OR ART OR HAART OR antiretroviral*) OR abstract:("Antiretroviral Therapy, Highly Active" OR ART OR HAART OR antiretroviral*))) OR abstract:((title:("Antiretroviral Therapy, Highly Active" OR ART OR HAART OR antiretroviral*) OR abstract:("Antiretroviral Therapy, Highly Active" OR ART OR HAART OR antiretroviral*)))) AND (title:("Medication Adherence" OR adherence OR "treatment adherence" OR "medication adherence" OR compliance) OR abstract:("Medication Adherence" OR adherence OR "treatment adherence" OR "medication adherence" OR compliance)) AND (title:("Viral Load" OR "viral suppression" OR "viral load suppression" OR "virologic suppression" OR "virologic failure") OR abstract:("Viral Load" OR "viral suppression" OR "viral load suppression" OR "virologic suppression" OR "virologic failure")) AND (title:("HIV Infections" OR HIV OR "people living with HIV" OR PLHIV) OR abstract:("HIV Infections" OR HIV OR "people living with HIV" OR PLHIV)) AND (title:(Ethiopia OR Ethiopian) OR abstract:(Ethiopia OR Ethiopian)) | 110 |
| Total |  |  | 1391 |
