# Supplementary material for: The effect of antiretroviral therapy adherence on viral load suppression rate among people living with HIV in Ethiopia: A systematic review and meta-analysis
Source: PLoS One. 2026 Sep 15;21(9):e0358352. doi: 10.1371/journal.pone.0358352 (PMC13577418; doi:10.1371/journal.pone.0358352)
Supplement: Supplementary file 8 [file pone.0358352.s004.docx]

Additional file 3: Quality Assessment of Included Studies Using the Adapted Newcastle-Ottawa Scale for Cross-Sectional Studies

| **Studies** | **Selection (Maximum 5 stars)** | | | | **Comparability (Maximum 2 stars)** | **Outcome (Maximum 3 stars)** | | **Total** | **Risk of bias** |
| --- | --- | --- | --- | --- | --- | --- | --- | --- | --- |
|  | **Representativeness of the sample** | **Sample size** | **Non-**  **respondents** | **Ascertainment of the exposure (risk factor** | **Confounding factors are controlled** | **Assessment of the outcome** | **Statistical test** |  |  |
| Abdullahi et al., 2020 | * | * | * | ** | ** | * | ** | 10 | Low |
| Abera et al, 2021 | * | * | * | ** | * | * | * | 8 | Low |
| Agegnehu et al., 2020 | * | * | * | ** | ** | * | ** | 10 | Low |
| Ahmed et al. , 2019 | * | * | * | ** | ** | * | ** | 10 | Low |
| Andarge et al., 2022 | * | * | * | ** | ** | * | ** | 10 | Low |
| Anito et al., 2022 | * | * | * | ** | ** | * | ** | 10 | Low |
| Atnafu et al., 2022 | * | * | * | ** | ** | * | ** | 10 | Low |
| Ayenew et al., 2024 | * | * | * | ** | ** | * | ** | 10 | Low |
| Belete et al., 2024 | * | * | * | ** | ** | * | ** | 10 | Low |
| Berhane et al. , 2024 | * | * | * | * | * | * | * | 7 | Low |
| Bisetegn et al., 2021 | * | * | * | ** | ** | * | ** | 10 | Low |
| Desalegn et al., 2024 | * | * | * | ** | ** | * | ** | 10 | Low |
| Alamneh et al., 2023 | * | * | * | ** | ** | * | ** | 10 | Low |
| Erjino et al., 2023 | * | * | * | ** | ** | * | ** | 10 | Low |
| Fentaw et al., 2020 | * | * | * | ** | ** | * | ** | 10 | Low |
| Gebrehiwot et al. , 2025 | * | * | * | ** | ** | * | ** | 8 | Low |
| Gedefa et al., 2025 | * | * | * | ** | ** | * | ** | 10 | Low |
| Gelaw et al., 2021 | * | * | * | ** | ** | * | ** | 10 | Low |
| Jaleta et al. , 2022 | * | * | * | ** | ** | * | ** | 10 | Low |
| Mamo et al., 2022 | * | * | * | ** | ** | * | ** | 10 | Low |
| Mehari et al., 2021 | * | * | * | ** | ** | * | ** | 10 | Low |
| Negash et al., 2020 | * | * | * | ** | ** | * | ** | 9 | Low |
| Muhie, 2024 | * | * | * | ** | ** | * | ** | 10 | Low |
| Nega et al. , 2020 | * | * | * | ** | ** | * | ** | 8 | Low |
| Seid et al., 2020 | * | * | * | ** | ** | * | ** | 10 | Low |
| Shumetie et al., 2021 | * | * | * | ** | * | * | ** | 8 | Low |
| Wedajo et al., 2021 | * | * | * | ** | ** | * | ** | 10 | Low |
| Zakaria et al., 2022 | * | * | * | ** | ** | * | ** | 10 | Low |
| Nuredin et al., 2025 | * | * | * | ** | ** | * | ** | 10 | Low |
| Abebe et al., 2024 | * | * | * | ** | ** | * | ** | 10 | Low |
| Diress et al., 2020 | * | * | * | ** | ** | * | ** | 8 | Low |
| Bogale et al., 2022 | * | * | * | ** | ** | * | ** | 10 | Low |
| Chirnet et al., 2024 | * | * | * | ** | ** | * | ** | 10 | Low |
| Emagnu et al., 2020 | * | * | * | ** | ** | * | ** | 10 | Low |
| Genet et al., 2021 | * | * | * | ** | ** | * | ** | 10 | Low |
| Kiros et al, 2022 | * | * | * | ** | ** | * | ** | 10 | Low |
| Lamesa et al., 2025 | * | * | * | ** | ** | * | ** | 10 | Low |
| Melaku et al., 2025 | * | * | * | ** | ** | * | ** | 10 | Low |
| Mena et al., 2023 | * | * | * | * | ** | * | * | 7 | Low |

**NEWCASTLE - OTTAWA QUALITY ASSESSMENT SCALE (Adapted for Cross Sectional Studies)**

**Selection: (Maximum 5 stars or 5 points)**

1) Representativeness of the sample:

a) Truly representative of the average in the target population. * (all subjects or random sampling): **1 point**
b) Somewhat representative of the average in the target population. * (non-random sampling) : **1 point**
c) Selected group of users: **0**
d) d) No description of the sampling strategy: 0

2) Sample size:

a) Justified and satisfactory: **1 point**
b) Not justified: **0**

3) Non-respondents:

a) Comparability between respondents and non-respondents’ characteristics is established, and the response rate is satisfactory: **1 point**
b) The response rate is unsatisfactory, or the comparability between respondents and non-respondents is unsatisfactory: **0**
c) No description of the response rate or the characteristics of the responders and the non-responders: **0**

4) Ascertainment of the exposure (risk factor):

a) Validated measurement tool: **(2points)**
b) Non-validated measurement tool, but the tool is available or described: **(1 point)**
c) No description of the measurement tool. **0**

**Comparability: (Maximum 2 stars or 2 points)**

1) The subjects in different outcome groups are comparable, based on the study design or analysis. Confounding factors are controlled.

a) The study controls for the most important factor (select one): 1 point
b) The study control for any additional factor: 1 point

**Outcome: (Maximum 3 stars or points)**

1) Assessment of the outcome:

a) Independent blind assessment: **2 points**
b) Record linkage: **2 points**
c) Self-report: **1 point**
d) No description: **0**

2) Statistical test:

a) The statistical test used to analyse the data is clearly described and appropriate, and the measurement of the association is presented, including confidence intervals and the probability level (p value): **1 point**
b) The statistical test is not appropriate, not described or incomplete. **0**
