# Supplementary material for: The effect of antiretroviral therapy adherence on viral load suppression rate among people living with HIV in Ethiopia: A systematic review and meta-analysis
Source: PLoS One. 2026 Sep 15;21(9):e0358352. doi: 10.1371/journal.pone.0358352 (PMC13577418; doi:10.1371/journal.pone.0358352)
Supplement: Supplementary file 9 [file pone.0358352.s010.docx]

**Response to Reviewers**

Dear Editor,

We sincerely thank you and the reviewers for the constructive comments and valuable suggestions on our manuscript entitled **“The effect of antiretroviral therapy adherence on viral load suppression rate among people living with HIV in Ethiopia: a systematic review and meta-analysis.”**

We have carefully addressed all comments and revised the manuscript accordingly. The revisions include clarification of adherence and viral load assessment timing, improved interpretation of regional and subgroup analyses, alignment of findings with the HIV care continuum, and acknowledgment of limitations related to heterogeneity and limited evidence in some subgroups.

We have also clarified the potential influence of ART regimen transitions, including the shift toward dolutegravir-based regimens in Ethiopia. However, due to insufficient regimen-specific reporting across included studies, we were unable to conduct subgroup analyses by regimen type or assess changes in viral suppression over time. This limitation has been acknowledged in the revised Discussion.

We appreciate the reviewers’ insightful feedback, which has helped improve the quality and clarity of our manuscript. Detailed responses to each comment are provided in the accompanying response letter.

Sincerely,

Desalegn Dawit Assele

On behalf of the co-authors

**Editor Comments:**

Introduction:

**Comment 1:** Consider shortening the section by removing information that is not directly related to the study objectives. This will make the section flow better.

***Response: Per suggestion, we have now polished the introduction section to improve conciseness and flow.***

**Comment 2:** Briefly, in your last paragraph, explain what knowledge gap this study intends to fill besides preexisting knowledge from previous studies.

***Response: Thank you for the insightful comment. Per suggestion, we have now polished the last paragraph of the introduction by incorporating an explanation of what knowledge gap the study intends to fill, besides preexisting knowledge from previous studies and clearly stating the objectives of the study.***

Methodology

**Comment 3:** To aid reproducibility, please include complete search strategy from at least one database, whether in the manuscript or as supplementary materials.

***Response: Thank you for this important suggestion. We agree that providing the complete search strategy enhances transparency and reproducibility of the systematic review. Accordingly, the detailed search strategy for the included databases has already been provided as Additional File 2 in the revised manuscript.***

Results:

**Comment 4:** Findings from the sensitivity analysis are repeated in some other subsections. Consider summarizing them concisely to aid clarity and to avoid confusion.

***Response: Thank you for this valuable suggestion. We have revised the sensitivity analysis sections throughout the results to improve clarity and reduce repetition. Repetitive interpretive statements were removed, while the key numerical findings and overall conclusions regarding the robustness of the results were retained. The revised text presents the sensitivity analyses more concisely and avoids redundancy with other subsections.***

Discussion:

**Comment 5:** Note that pooled estimates are based on observational studies and hence findings cannot be interpreted as causality. For example, the association between adherence and VL suppression should be interpreted with caution.

***Response: Thank you for this insightful suggestion. We have now revised and made the necessary corrections to the discussion and conclusion parts to frame our interpretation within the scope of our study.***

Conclusion:

**Comment 6:** Closely related to the comment above, avoid saying adherence "significantly increases VL suppression. something like "good adherence was strongly associated with VL suppression" will be safer.

***Response: Thank you for this insightful suggestion. We have now revised and made the necessary corrections to the discussion and conclusion parts to frame our interpretation within the scope of our study.***

**Reviewer #1:**

**Comment 1:** I have noted that the manuscript has merit to be published in Plos One journal. However, the authors need to address the minor comments given in the attachment line-by-line. The authors may need to carefully format their MS as per the author guidelines.

***Response: We sincerely thank the reviewer for the positive evaluation of our manuscript and for recognizing its potential contribution to the literature. We have carefully reviewed and addressed all comments provided in the attachment on a point-by-point basis. Each suggestion has been considered, and appropriate revisions have been made throughout the manuscript to improve clarity, completeness, and scientific rigor. In addition, we have carefully revised the manuscript formatting, structure, and presentation to ensure compliance with the PLOS ONE author guidelines.***

**Comment 2:** Overall, the manuscript is well written and thoughtfully structured. However, several minor issues require attention before publication. These include inconsistent use of abbreviations after their initial definition, typographical errors (e.g., "art" instead of "ART"), and other minor editorial and formatting issues that should be carefully reviewed and corrected throughout the manuscript.

***Response:*** ***We sincerely thank the reviewer for the constructive feedback and careful review of our manuscript. We have thoroughly revised the manuscript to ensure consistent use of abbreviations after their initial definition, corrected typographical errors (including capitalization of "ART"), and addressed other grammatical, editorial, and formatting issues throughout the text. We have also carefully reviewed the manuscript to improve consistency, clarity, and adherence to the PLOS ONE formatting requirements.***

**Comment 3:** On page 5, the eligibility criteria define good ART adherence using multiple measurement approaches (e.g., pill counts, pharmacy refill records, self-report, and electronic monitoring). It is unclear whether all included studies used these methods or whether adherence measurement varied across studies. If variability existed, this should be explicitly described. Additionally, self-reported adherence is subject to recall and social desirability bias; therefore, it would be preferable to specify the actual adherence measurement methods used in the included studies rather than listing all possible approaches.

***Response: We thank the reviewer for this important observation. We agree that the previous description was overly broad and could create ambiguity regarding the actual adherence measurement methods used in the included studies. We have revised the eligibility criteria to specify the adherence assessment approach applied in the included studies. Specifically, good ART adherence was defined as taking at least 95% of their prescribed ART doses (or < 3 missed doses per month for a once-daily regimen) based on self-reported adherence****,* ***as reported by the individual studies. We have also acknowledged the potential limitations of self-reported adherence, including recall and social desirability bias, in the limitations section of the manuscript.***

**Comment 4:** Did the included studies consider the timing of adherence assessment relative to viral load testing? Specifically, was adherence measured before, concurrent with, or after viral load assessment? Any variability in the timing of adherence measurement across studies should be clearly described, as it may affect the interpretation of the association between adherence and viral suppression.

***Response: Thank you for this important comment. We agree that the timing of adherence assessment relative to viral load testing may influence the interpretation of the association. We have clarified this aspect in the revised manuscript. Most included studies assessed adherence before or at the time of viral load measurement; however, the interval between adherence assessment and viral load testing varied, and some studies did not clearly report the timing. This variability has been acknowledged as a potential source of heterogeneity and limitation in interpreting the pooled findings.***

**Comment 5:** WHO guidelines, adopted by Ethiopia and many other countries, recommend viral load testing at 6 months after ART initiation, at 12 months, and annually thereafter. Since viral load suppression may vary across these time points, it is important to clarify whether the timing of viral load assessment was considered during study selection or analysis. If this factor was controlled for, it should be explicitly described in the methods section, as differences in testing intervals may contribute to heterogeneity across studies. At list this issue needs to be acknowledged in the discussion.

***Response: Thank you for this valuable comment. We agree that the timing of viral load assessment may influence viral suppression outcomes and contribute to heterogeneity across studies. We have clarified this issue in the revised manuscript. The included studies reported viral load measurements at different time points following ART initiation, and this variability was considered during data extraction. However, due to limited reporting and heterogeneity in study designs, we were unable to perform subgroup analysis based on viral load assessment timing. This limitation has been acknowledged in the Discussion section.:***

**Comment 6:** On pages 10–11, the subgroup analysis indicates that:

i. Children achieved higher viral load suppression than adults. This finding appears inconsistent with many published studies and routine program data, which generally report lower suppression among children. The authors should provide a stronger explanation for this observation and explore potential reasons

***Response: Thank you for this keen observation. As you stated, several previous studies (particularly before 2017) generally report lower suppression among children. Similarly, two recent systematic reviews assessing virologic suppression reported comparable rates of suppression between children and adults. Accordingly, we have now incorporated a potential explanation for this observation and explored potential reasons in the discussion section.***

ii. Given the substantial variation in the number of studies across regions, regional comparisons of VLS should be interpreted with caution. In particular, findings for Tigray, based on only two studies, may be unreliable and potentially misleading. Consider toning down the interpretation for Tigray and focusing on regions represented by at least three studies.

***Response: Thank you for this important comment. We agree that regional comparisons based on a small number of studies should be interpreted cautiously. We have revised the interpretation by avoiding strong conclusions for regions represented by fewer studies, particularly Tigray (n=2), and have emphasized findings from regions with adequate study representation (≥3 studies). We have also acknowledged the limited number of studies per region as a potential source of uncertainty in the Discussion section.:***

iii. The findings related to regimen type are also inconsistent with existing evidence and therefore require stronger justification and clearer interpretation. The authors should provide a more detailed explanation for these results, considering possible factors such as differences in patient characteristics, prior treatment failure, adherence patterns, and the composition of the included studies.

***Response: Thank you for this insightful comment. We agree with the comment, and this observation requires stronger justification and clearer interpretation. Accordingly, we have now added a dedicated paragraph in the discussion section addressing this issue.***

**Comment 7:** Minor suggestion: Consider presenting the findings in the order of the HIV care continuum, consistent with the title of your review (i.e., adherence followed by viral load suppression throughout the MS).

***Response: Thank you for this helpful suggestion. We have revised the manuscript to improve consistency with the review title and the HIV care continuum framework by presenting adherence-related findings before viral load suppression outcomes throughout the manuscript.:***

**Comment 8:** In P2 under the discussion section: Access to viral load testing may not adequately explain the observed differences, as this review included treatment-experienced PLHIV who had undergone viral load testing. This explanation is more applicable to population-level VLS studies than to ART cohort studies and should therefore be reconsidered.

***Response: Thank you for this insightful comment. As suggested, access to viral load testing doesn’t adequately explain the observed differences since participants have undergone viral load testing at least once. However, we used the phrase “access to routine viral load monitoring”, which may contribute to improved virologic suppression by enabling early detection of treatment failure, facilitating timely adherence interventions and regimen optimization, and preventing prolonged viremia and drug resistance.” We believed this fine difference is particularly important in our resource-limited setup, where most facilities do viral load testing as frequently as recommended due to resource limitations. Overall, a multitude of factors potentially contribute to the observed differences, including appropriate treatment response monitoring, and per suggestion, we have polished the specific paragraph to improve clarity of our explanation.***

**Comment 9:** In the same paragraph, the low VLS rate in Ethiopia is attributed to recent funding cuts and their impact on the HIV response. This interpretation requires stronger supporting evidence. Do studies conducted before the funding cuts support this argument? Moreover, gaps in viral load testing are not the focus of this review and do not adequately explain VLS among treatment-experienced PLHIV. Given that VLS in Ethiopia has generally improved over time and is approaching global targets, any explanation for the observed findings should be evidence-based and appropriately contextualized to strengthen the manuscript and its relevance for the national HIV program.

***Response: Thank you for this important comment. We agree that VLS in Ethiopia has generally improved over time and is approaching global targets. However, the VLS rate still lags behind the global target. In the third paragraph of the Discussion (“Potential reasons for this discrepancy from the globally set targets include…”), we mainly focus on highlighting potential factors contributing to this gap with global targets. Studies have shown that several factors contribute to the lower rates of VLS observed in low-resource countries such as Ethiopia, including limitations in the HIV response and the inability to achieve full-scale programmatic implementation, which may contribute to the lag behind global targets. The impact of recent funding cuts on the HIV response has also been supported by evidence from low-resource countries. We have addressed the comment regarding viral load testing in our response to the previous comment. Overall, a multitude of factors may contribute to the observed differences, including resource availability and appropriate treatment-response monitoring. We have therefore revised the paragraph to provide a more evidence-based and appropriately contextualized discussion of factors relevant to VLS in Ethiopia.***

**Comment 10:** Regarding subgroup analysis (discussion section P3)

I. As noted in Comment 5(ii), caution is warranted when interpreting findings from regions represented by only a few studies. The discussion should acknowledge this limitation and avoid drawing strong conclusions from subgroups with limited evidence.

***Response: Thank you for this important observation. We have revised the Discussion section to acknowledge the limited number of studies contributing to some regional subgroup analyses and have moderated the interpretation of findings from regions with sparse evidence. We now emphasize that regional differences should be interpreted cautiously due to potential uncertainty arising from limited study representation.:***

II. I did not find pregnant women included in the subgroup analysis, although they are discussed later in the manuscript. The supplementary table indicates that one study involving pregnant women was included. Please revisit this section to ensure consistency between the included studies, subgroup analysis, and discussion, or clarify why this study was not incorporated into the subgroup analysis.

***Response: Thank you for identifying this inconsistency. We have reviewed the subgroup analysis and revised the manuscript to ensure consistency between the included studies, subgroup analyses, and discussion. The study involving pregnant women was included in the review; however, it was not included as a separate subgroup analysis due to the limited number of studies available (only one study). This rationale has now been clarified in the revised manuscript.:***

III. Similar comments to #8 on subgroup analysis for adherence.

***Response: Thank you for this comment. We have reviewed the subgroup analysis for ART adherence and revised the manuscript to ensure consistency between the included studies, subgroup analyses, and discussion. Subgroup findings based on a small number of studies have been interpreted cautiously, and we have avoided drawing strong conclusions where the available evidence was limited. The limitations related to sparse subgroup data and potential uncertainty have also been acknowledged in the Discussion section.***

**Reviewer #2:**

It is a good study, however, it would have been interesting to know if adherence rates varied based on the type of regimen and even what the first-line regimen in Ethiopia is, as it is reported that some regimens, such as the dolutegravir based, have greater adherence due to the better side effect profile compared to mixed or second-line regimens. In addition, was there any change in first-line regimen from 2019 to 2025, as some countries transitioned to dolutegravir-based regimens at this time, and if there was a change in regimen across the years, was there an observed change in viral suppression due to this change?

***Response: Thank you for this important comment. We agree that ART regimen type, particularly the transition to dolutegravir (DTG)-based regimens, may influence adherence and viral suppression outcomes. We have added information on Ethiopia’s ART regimen transition and clarified this aspect in the revised manuscript. However, due to limited reporting of regimen-specific adherence and viral suppression outcomes across the included studies, we were unable to perform subgroup analyses by ART regimen type or evaluate changes in viral suppression associated with regimen transitions over time. We have acknowledged this as an important evidence gap and limitation in the Discussion section, and highlighted the need for future studies to assess regimen-specific treatment outcomes.***
